# SciSt: single-cell reference-informed spatial gene expression prediction from pathological images

**DOI:** 10.1093/bib/bbaf613

**Published:** 2025-11-20

**Authors:** Yixin Li, Fan Zhong, Lei Liu

**Affiliations:** Institutes of Biomedical Sciences, Fudan University, 130 Dong'an Road, Xuhui District, Shanghai 200032, China; Intelligent Medicine Institute, Fudan University, 138 Yixueyuan Road, Xuhui District, Shanghai 200032 China; Intelligent Medicine Institute, Fudan University, 138 Yixueyuan Road, Xuhui District, Shanghai 200032 China; Shanghai Institute of Stem Cell Research and Clinical Translation, Shanghai East Hospital, Tongji University, 1500 Yuntai Road, Pudong New Area, Shanghai 200120, China; Shanghai Institute of Infectious Disease and Biosecurity, Fudan University, Shanghai 200032, 130 Dong'an Road, Xuhui District, China

**Keywords:** deep learning, spatial gene expression, single-cell reference, pathological images

## Abstract

The widespread application of spatial transcriptomics in uncovering disease mechanisms remains limited by the scarcity of samples and the high experimental costs, which have not declined substantially in recent years. Unlocking the vast resources of clinical H&E-stained images could provide an efficient and cost-effective alternative for large-scale spatial analysis. However, predicting spatial gene expression from histopathological images remains challenging, as existing end-to-end frameworks often fail to capture the intrinsic transcriptomic structures observed in real transcriptomics data. To address this, we developed SciSt, a deep learning framework that predicts spatial gene expression by integrating pathological features with biologically informed initial gene expressions. These initial expressions are generated through a weighted strategy combining cell segmentation and single-cell reference data, thereby enhancing biological interpretability. SciSt achieved state-of-the-art performance across three benchmark datasets, outperforming the second-best models by 21.4% and 13.7%, respectively, and demonstrated robust generalization on the TCGA-BRCA and TCGA-LIHC cohorts. Beyond accurate prediction, SciSt enables cross-modal translation between morphology and gene expression, offering new avenues for mining the untapped potential of clinical image archives. This work highlights how prior biological knowledge can substantially advance the interpretability and scalability of biomedical AI models.

## Introduction

Spatial transcriptomics (STs) is an emerging technology with great potential to uncover disease mechanisms and advance precision medicine [1]. By jointly capturing gene expression and spatial localization within tissues, ST provides unprecedented insights into cellular organization and molecular pathology [2]. However, its adoption is limited by high experimental costs and restricted sample availability [3]. Meanwhile, vast repositories of H&E-stained histopathological slides remain underutilized, representing an untapped resource for large-scale spatial analysis. As experimental costs are unlikely to decrease substantially in the near term, developing computational approaches that infer spatial gene expression from pathology images is essential to unlock the full potential of clinical image archives.

Pathology images provide cost-effective cellular detail, including nuclear morphology and tissue heterogeneity, essential for disease characterization and clinical assessment [4]. Cross-modal studies have begun to infer phenotypes from such images [5–7], and several works predict gene expression from whole-slide images [8], yet most remain slide level or single gene [9], weakening morphology–transcriptome correspondence. With advances in deep learning and computer vision, spatial multigene prediction directly from pathology images has become feasible [10, 11]. The task is to align high-resolution pathology with STs, use sequencing as ground truth, and learn a spot-level mapping from image features to multigene expression, enabling spatially resolved prediction without additional sequencing.

Several studies have investigated spatial multigene expression prediction from pathology images. ST-Net [12] employs convolutional neural networks (CNNs) to capture morphological patterns. HisToGene [13] and His2ST [14] incorporate positional information via Vision Transformer (ViT) sequence embeddings and CNN–GCN integration to model global and neighborhood context. To better represent high-resolution structures, THItoGene [15] integrates positional and multiview histological features, yet relies on fixed input granularity. More recently, TCGN [16] combines Transformers with convolution and graph-based co-embedding and reports state-of-the-art (SOTA) performance, while incurring higher computational and memory costs that may affect scalability.

Despite increasing model complexity, gains in predictive performance remain limited. We therefore propose a biology-informed framework that models each spot’s initial expression (IE) as a mixture of its constituent cell types [17]. The method first estimates per-spot cell-type composition via nucleus segmentation and cell-type classification [18], and then uses single-cell transcriptomic references [19] to map the estimated cell counts to an initial multigene expression profile for each spot. This profile serves as a conditioning prompt during training. By shifting emphasis away from increasingly deep end-to-end architectures and grounding predictions in cellular priors, the framework improves interpretability, data efficiency, robustness, and scalability.

**Figure 1:**
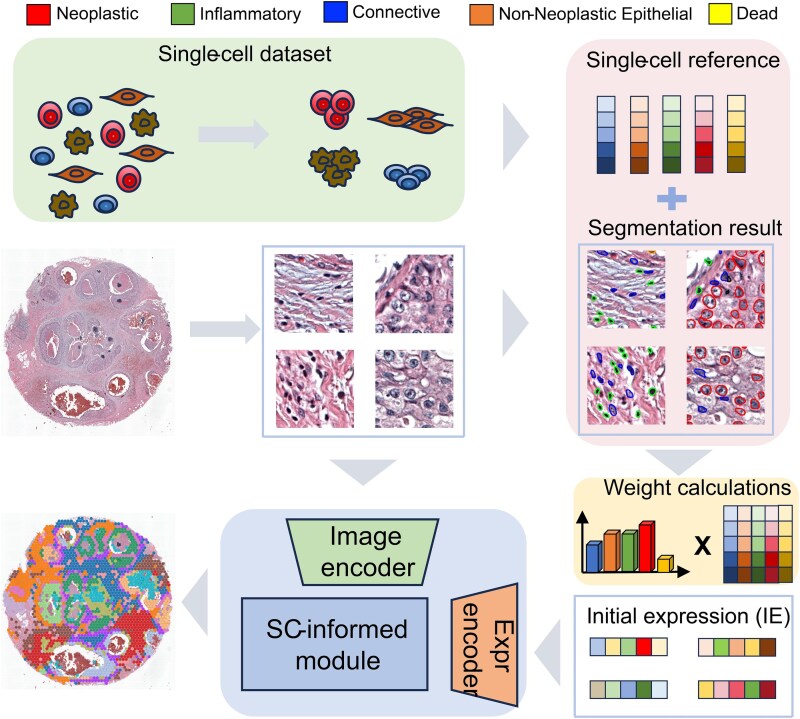
Workflow of the study. The single-cell dataset was used as a reference for generating IE and organized by the cell types, which involved neoplastic cells, inflammatory cells, connective cells, non-neoplastic epithelial cells, and dead cells. The reference matrix and patch segmentation results from the pathological images were multiplied by weight to construct the IE. Then, the patch and IE were fed into SciSt to output gene expression prediction results for the whole image. SciSt consisted of three modules: image encoder, expression encoder, and SCinformed module.

We present SciSt, a single-cell–informed framework that implements this design (Fig. 1). SciSt attains SOTA accuracy with lower architectural complexity. Beyond accuracy, SciSt provides a reproducible and modular pipeline that advance integration between pathology and single-cell transcriptomics, laying groundwork for future multimodal spatial biology. Its main limitation is a focus on morphology-linked expression, with reduced sensitivity to signals not evident in tissue morphology.

## Materials and methods

### Workflow of the study

SciSt integrates histopathology features with single-cell references to improve spatial gene expression prediction. For each image patch, we count cells by type and derive weights that, combined with scRNA-seq type prototypes, yield a SC-informed IE. An image encoder and an expression encoder embed the patch and IE, and an SC-informed module predicts gene expression for a predefined gene set. The scRNA-seq reference is fixed per cancer type, while pathology images are flexible inference inputs. We hypothesize that IE provides biologically grounded priors that enhance prediction. The workflow is shown in Fig. 1, and the definition of symbols if provided in Table S2.

### Spatial transcriptomic datasets and processing

We incorporated the human epidermal growth factor receptor 2 positive breast cancer (HER2+) dataset [20], the cutaneous squamous cell carcinoma (cSCC) dataset [21], the Pancreas cancer (PAAD) dataset [22]， the breast cancer dataset [12], and the 10× Genomics liver cancer dataset (Table S1). These are all ST datasets with high-resolution Whole Slide Image (WSI), providing precise transcriptomics and spot locations. The first three datasets were used to compare the performance of SciSt with previous SOTA models, and the rest of the datasets were applied to validate the generalizability of SciSt in bulk RNA-seq data.

The preprocess of ST datasets involves two steps: image and gene expression. We split the WSIs into $224\times 224$ patches by extending spot center coordinates 112 pixels along with *x* and *y* axis directions, enabling the primary view of patches consistent with the spots.

Given the spatial gene expression data ${\mathrm{G}}_{\mathrm{ST}}$ from an ST dataset, the subset of genes to be predicted, denoted as $\mathrm{G}$, was selected based on two criteria:

i) The genes exhibited high variance across all WSIs;
ii) They were also sequenced in the corresponding single-cell reference ($\mathrm{SCR}$) dataset.

The expression values of genes in $\mathrm{G}$ were normalized and log-transformed at the spot level using the Python package Scprep, and these processed values were used as the labels for model training. To ensure data quality, samples containing fewer than 180 nonbackground spots were excluded, consistent with the filtering strategy used in previous studies.

### Cell segmentation and inference configuration

We segmented and classified cells in each pathology patch using Hover-Net [23] trained on the PanNuke dataset [24]. PanNuke provides five coarse cell types: neoplastic, inflammatory, connective, non-neoplastic epithelial, and dead. Let $C$ be this set of cell types, and let ${N}_{c_i}$ denote the number of cells of type ${c}_i\in C$ detected in a patch. The total cell count for a patch is therefore ${N}_{total}={\Sigma}_{c_i\in C}{N}_{c_i}$. This cell-type inventory provides biological context for the images and allows us to initialize the per-patch IE. These coarse-grained categories are adaptable to diverse single-cell transcriptomic datasets, enabling patch-to-reference mapping and defining IE at the level of major cell types.

### Single-cell reference datasets and processing

We hypothesize that within the same tumor type, cell-type–specific gene-expression profiles differ only slightly. Therefore, ST datasets from that tumor type can share a $\mathrm{SCR}$. The $\mathrm{SCR}$ provides a canonical gene-expression vector ${\mathrm{Ref}}_{c_i}$ for each cell type ${c}_i$. We combined these references with the corresponding cell-type counts ${N}_{c_i}$ to initialize the patch-level IE vector over $\mathrm{G}$.

#### Cell type selection criteria for ${c}_i$ in $\mathrm{SCR}$

Because the composition of major and minor cell types varies across tumor types, we selected a representative profile for each ${c}_i$ using two criteria: (i) when choosing major cell-type groups, we prioritized those with fewer minor subtypes to reduce intra-class heterogeneity; (ii) within a major group, we selected the minor subtype with the largest number of cells. Cells classified as “dead” were excluded from all computations.

#### Constructing gene-expression references for $\mathrm{G}$

For each ${c}_i$, we collected all single cells assigned to ${c}_i$ in the $\mathrm{SCR}$ to form a matrix ${\mathrm{Ref}}_{c_i}\in{\mathbb{R}}^{N_{\mathrm{mc}}^i\times{N}_{\mathrm{G}}}$, where ${N}_{\mathrm{mc}}^i$ is the number of single cells for ${c}_i$ and ${N}_{\mathrm{G}}$ is the number of $\mathrm{G}$. We averaged across cells (the first dimension) to obtain ${\mathrm{Ref}}_{c_i}\in{\mathbb{R}}^{N_{\mathrm{G}}}$, followed by normalization and logarithmic transform to yield the final reference vector for ${c}_i$.

### Architecture of SciSt

Similar to the segment anything model (SAM) [25], SciSt comprises two encoders and one decoder. We refer to the decoder as the SC–informed module. In this architecture, the IE acts as a prompt that guides the final prediction. The two encoders project their respective inputs into a shared latent space, and the SC–informed module fuses these representations to produce the output.

#### Construction of initial expression and expr encoder

Given reference vectors ${\mathrm{Ref}}_{c_i}$ and cell counts ${N}_{c_i}$ for cell type ${c}_i\in C$, per-patch expression vector is a weighted average:


$$ \mathrm{Expr}={\Sigma}_{c_i\in C}\left({\pi}_i{\mathrm{Ref}}_{c_i}\right) $$

$$ {\pi}_i=\frac{N_{c_i}}{\Sigma_{c_i\in C}{N}_{c_i}} $$

The expression encoder consists of linear layers with a GELU activation:


$$ \mathrm{Expr}\ \mathrm{embedding}=\mathrm{Linear}\left(\mathrm{GELU}\left(\mathrm{Linear}\left(\mathrm{Expr}\right)\right)\right) $$

$$ \mathrm{Expr}\ \mathrm{embedding}\in{\mathbb{R}}^{1\times{d}_{att}} $$

The attention dimension ${d}_{att}$ matches the channel dimension of the image encoder’s final map (512 for ResNet34).

#### Construction of image encoder

The image encoder we employed in this study was CNN-SASM [26] (Fig. S1), which combines a CNN backbone with channel and spatial self-attention. The CNN is a lightweight ResNet34 truncated at the last two layers and initialized with ImageNet pretraining. Let ${X}_{\mathrm{img}}$ be the input image patch:


$$ \mathrm{Latent}\_{\mathrm{Vector}}_{\mathrm{Image}}=\mathrm{SSA}\left(\mathrm{CSA}\left(\mathrm{CNN}\_\mathrm{module}\left({X}_{\mathrm{img}}\right)\right)\right) $$

$$ \mathrm{Latent}\_{\mathrm{Vector}}_{\mathrm{Image}}\in{\mathbb{R}}^{N_{\mathrm{coord}}\times{d}_{att}} $$

Here CSA and SSA denote channel and spatial self-attention layers. ${N}_{\mathrm{coord}}=H\times W$ is the number of spatial positions in the final CNN feature map. We use ${N}_{\mathrm{coord}}=49$.

#### Construction of SC-informed module

**Figure 2:**
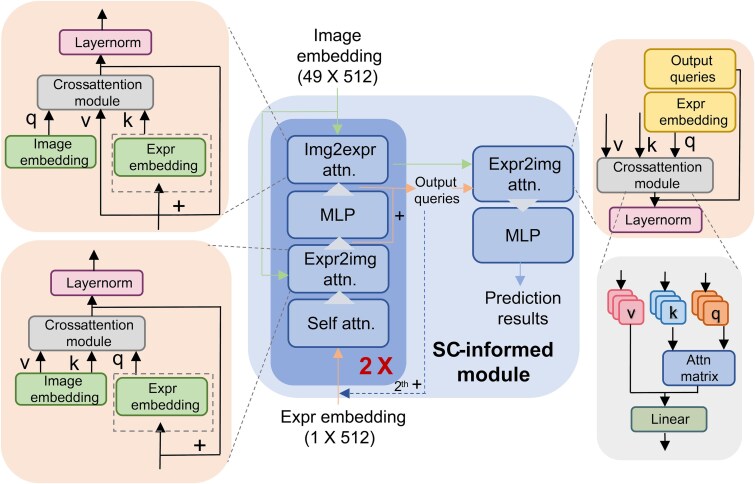
The detailed architecture of the SC-informed module. The SC-informed module is a double-ended input frame with image embedding and expr embedding. The output queries are the middle outputs in the SC-informed module. The main computing modules contain one self-attention module, two expr2img attention modules, two MLP modules, and one img2expr module. The part that is repeated twice during training is highlighted.

The SC-informed module enables the IE to guide image embeddings. Its design follows the spirit of the SAM light decoder while replacing prompt tokens with the expression embedding and removing the upsampling and mask heads. The goal is to accelerate training and improve accuracy by injecting expression priors directly into the decoding stage. The SC-informed module had six primary parts (Fig. 2): one self-attention module, two expr2img attention modules, one img2expr attention module, and two MLP modules.

The expr2img attention module implemented guidance from the expression embedding to the image tokens, whereas the img2expr attention module performs the reverse. The two differ only in how they set the query, key, and value. As for the former, the query is formed by adding the expression embedding to the output of the self-attention module, and the image embedding serve as both key and value for computing the correlation scores .


\begin{align*} &{\mathrm{Output}}_{\mathrm{expr}2\mathrm{img}}\\ & \quad =\mathrm{Attention}\ \left(q=\mathrm{Sum}\left(\mathrm{Expr}\ \mathrm{embedding},{\mathrm{Output}}_{\mathrm{self}\hbox{-}\mathrm{attention}}\right)\right.\\&\quad\ \, k=\mathrm{Image}\ \mathrm{embedding}\\&\quad \left. v=\mathrm{Image}\ \mathrm{embedding}\right) \end{align*}

The query, key, and value of the img2expr module were as follows:


\begin{align*} &{\mathrm{Output}}_{\mathrm{img}2\mathrm{expr}} =\mathrm{Attention}\ \left(q=\mathrm{Image}\ \mathrm{embedding}\right. \\& \quad \ \ k=\mathrm{Sum}\left(\mathrm{Expr}\ \mathrm{embedding},{\mathrm{Output}}_{\mathrm{MLP}}\right)\\&\quad\ \,\left. v={\mathrm{Output}}_{\mathrm{MLP}}\right) \end{align*}

These cross-attention steps tightly integrate the image and expression embeddings. The final expr2img attention module makes the expression embedding the dominant signal in the decoder’s output. Noatbly, in the second iteration we re-injecte the intermediate MLP output alongside the expression embedding and use this combination to initialize the subsequent input, which improves training flexibility and effectiveness. In the first iteration, the query is initialized with the expression embedding alone. The complete data flow is shown in Fig. 2.


$$ \left\{\begin{array}{@{}ll}\mathrm{Output}\ \mathrm{queries}=\mathrm{Expr}\ \mathrm{embedding} & \mathrm{if}\ \mathrm{iteration}\ \mathrm{round}=1\\{}\mathrm{Output}\ \mathrm{queries}={\mathrm{Output}}_{\mathrm{MLP}} & \mathrm{if}\ \mathrm{iteration}\ \mathrm{round}=2\end{array}\right. $$

Training uses a batch size of 32 for 81 epochs with mean-squared error as the loss and Adam as the optimizer. The same hyperparameters are applied to the compared models.


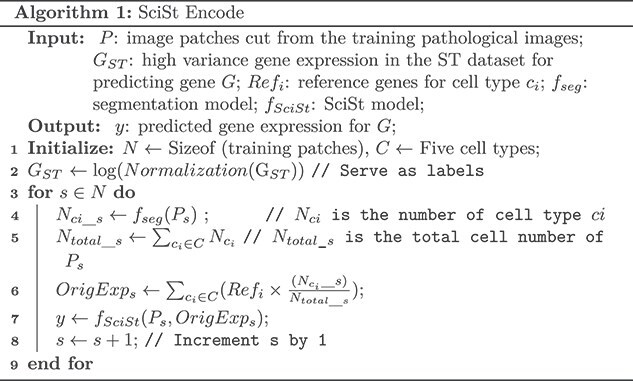


## Results

### SciSt predicts gene expression more accurately than SOTA models

Our study primarily benchmarked against the SOTA models TCGN and THItoGene, and we additionally included HisToGene as a supplementary baseline. Experiments were conducted on the HER2+, cSCC, and PAAD datasets using leave-one-section-out cross-validation, consistent with prior work.

**Figure 3:**
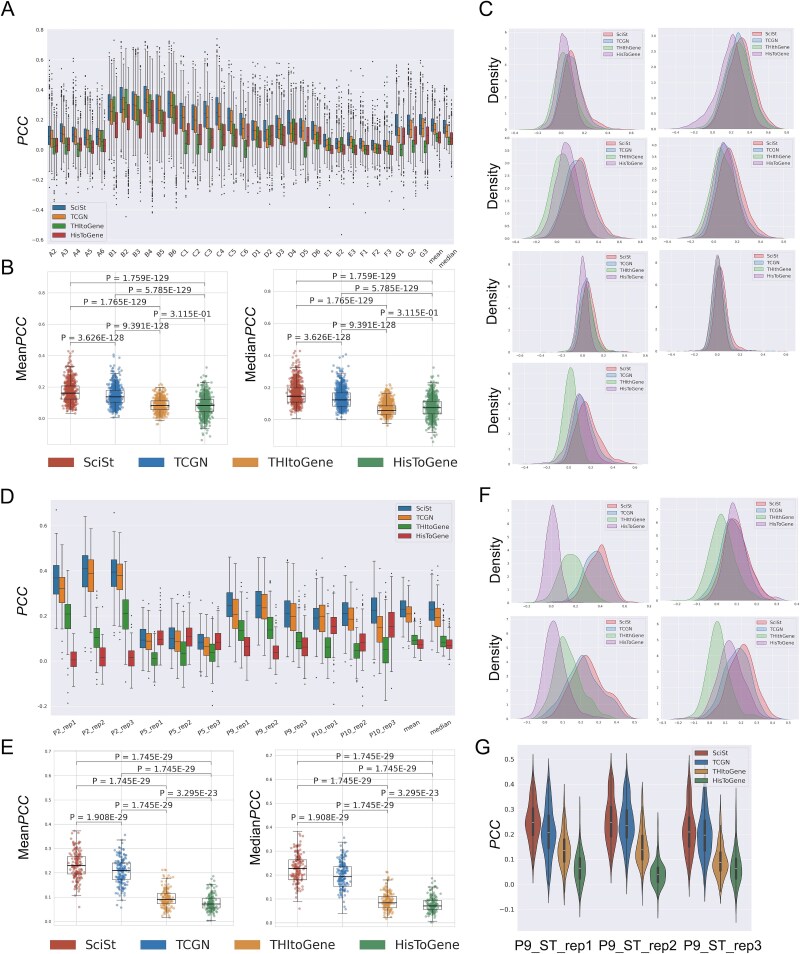
The performance of SciSt compared to other SOTA models on HER2+ and cSCC datasets. (A) The comparison of SciSt with the other SOTA models on the HER2+ dataset. The evaluation indicator was the PCC of all genes belonging to the specific sample. Mean and median PCC were calculated across all samples to assess the overall performance of models. (B) Wilcoxon sign-rank tests for mean and median PCC of three models on the HER2+ dataset. (C) Kernel density plots of three models’ gene PCC on different patients of the HER2+ dataset. The PCC for each patient was generated by averaging the PCC of all samples belonging to the patient. The bulged area on the right corresponds to the well-predicted genes by SciSt. (D) The comparison of SciSt with other previous models on the cSCC dataset. (E) Wilcoxon sign-rank tests for mean and median PCC of three models on the cSCC dataset. (F) Kernel density plots of three models’ gene PCC on different patients of the cSCC dataset. (G) Violin plot of P9_ST_rep1 sample’s PCC on the cSCC dataset.

Across HER2+, cSCC, and PAAD, SciSt achieved the highest per-sample *PCC*s (Fig. 3A, D, Fig. S4), and the best mean and median *PCC* (Fig. 3B, E, Fig. S4B). Pairwise Wilcoxon signed-rank tests on mean and median *PCC* showed SciSt significantly outperformed the other models in all three datasets (*P* < .001). In HER2+, SciSt’s mean *PCC* was 0.169 (95% CI 0.164-0.173), 0.025 higher than TCGN, the second-ranked model. Detailed information for the cSCC and PAAD datasets can be found in Table 1.

**Table 1:** The overall performance of SciSt compared to other SOTA models.

Dataset	Model	Mean *PCC*		Median *PCC*	
		Average	95% CI	Average	95% CI
HER2+	SciSt	0.169	0.164–0.173	0.159	0.154–0.163
	TCGN	0.144	0.140–0.148	0.131	0.126–0.135
	THItoGene	0.085	0.082–0.088	0.063	0.060–0.065
	HisToGene	0.082	0.078–0.086	0.077	0.073–0.082
cSCC	SciSt	0.231	0.223–0.240	0.224	0.215–0.234
	TCGN	0.206	0.198–0.214	0.197	0.188–0.207
	THItoGene	0.096	0.090–0.102	0.090	0.084–0.097
	HisToGene	0.077	0.072–0.082	0.076	0.071–0.082
	SciSt	0.317	0.292–0.341	0.316	0.290–0.342
PAAD	TCGN	0.260	0.234–0.286	0.256	0.227–0.284
	THItoGene	0.015	0.001–0.029	0.019	0.004–0.034
	HisToGene	0.164	0.139–0.188	0.166	0.143–0.188

Additionally, SciSt yielded substantially more well-predicted genes than the other models across samples (Fig. 3C, F, Table S6). Notably, the P9 patient’s violin plot showed a distinct inverted-triangle pattern, indicating a cluster of highly predicted genes, not evident in the other models (Fig. 3G). Violin plots for the remaining patients are provided in Fig. S2. Consistently, Fig. S3A presents the same trend when expressed as counts of highly predicted genes. Beyond counts, dispersion scores (supplementary methods), measuring the magnitude of high-*PCC* deviations from the sample median, also followed this pattern: SciSt generally achieved the highest dispersion across most samples (Fig. S3B).

SciSt had the best computational efficacy and advantages in the training process, such as GPU memory usage and runtime (Table 2). Although SciSt had one more input data than TCGN, the IE was sourced directly from the spot image.

**Table 2:** The basic configuration of SciSt compared to other SOTA models during training.

Model	Input data	Batch size	Number of parameters	GPU memory	Time/epoch
SciSt	Image and IE	32 (default)	34.07 M	4067 MiB	16 min
		16		1788 MiB	26 min
		64		4832 MiB	7 min
TCGN	Image	32 (default)	22.05 M	7629 MiB	30 min
		16		3434 MiB	37 min
		64		11,860 MiB	17 min
THItoGene	Image and coordinates	1 (default)	47.67 M	/	2 min
HisToGene	Image and coordinates	1 (default)	106.52 M	/	0.08 min

Note: The comparison of GPU memory usage on models was based on the same batch size. The THItoGene and HisToGene models did not have the GPU memory parameter because their inputs are in samples rather than spot images, which is inconsistent with the other two models. The batch size had to be fixed at 1 for their comparison; time/epoch was tested on the HER2+ dataset.

### Spatial visualization of top accuracy genes

To identify accurately predicted genes, we computed per-gene adjusted *P*-values under a null of no positive correlation between predicted and observed expression. The top four genes with the lowest *P*-value in the HER2+ dataset were *C3*, *FASN*, *GNAS*, and *IGHA1*, while in the cSCC dataset, they were *RPL13*, *SUMF2*, *PTP4A2*, and *IMP4*. Prior studies link *FASN*, *GNAS*, and *IGHA1* to breast cancer progression and suggest prognostic potential [27–29]. For cSCC, no direct links to progression have been reported. However, *SUMF2*, *PTP4A2*, and *IMP4* are associated with other types of carcinomas [30–32].

**Figure 4:**
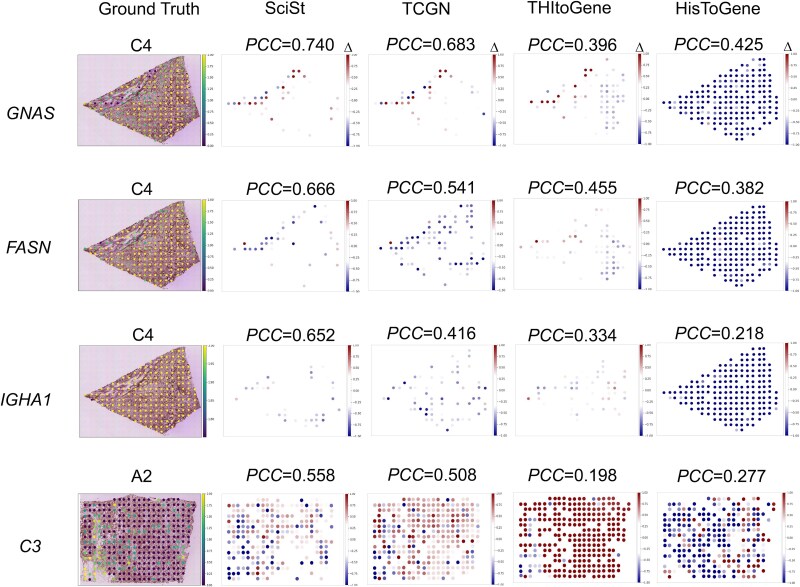
Spatial visualization of top accurately predicted genes on the HER2+ dataset. Top accurately predicted genes were selected according to the positive correlation significance tests, including GNAS, FASN, IGHA1, and C3. We visualized the spatial distribution of the differences between these predicted gene expressions and the ground truths on the sample with the highest gene PCC. Spots indicating positive and negative deviation correspond to predicted expression higher or lower than the ground truth, respectively, while spots within a predefined tolerance are considered acceptable. A common deviation scale is used across all panels.

Then, we visualized the spatial distribution of four top genes (Fig. 4). SciSt showed the largest proportion of low-error (white) regions, suggesting its more accurate absolute predictions. Similar results could also be verified on the cSCC dataset (Fig. S5). Quantitatively, SciSt achieved the highest *PCC* on each sample. Remarkably, the *PCC* for the *GNAS* gene reached 0.740, the highest value among all reported studies.

### Spatial visualization of leukocyte mediated immunity gene set

**Figure 5:**
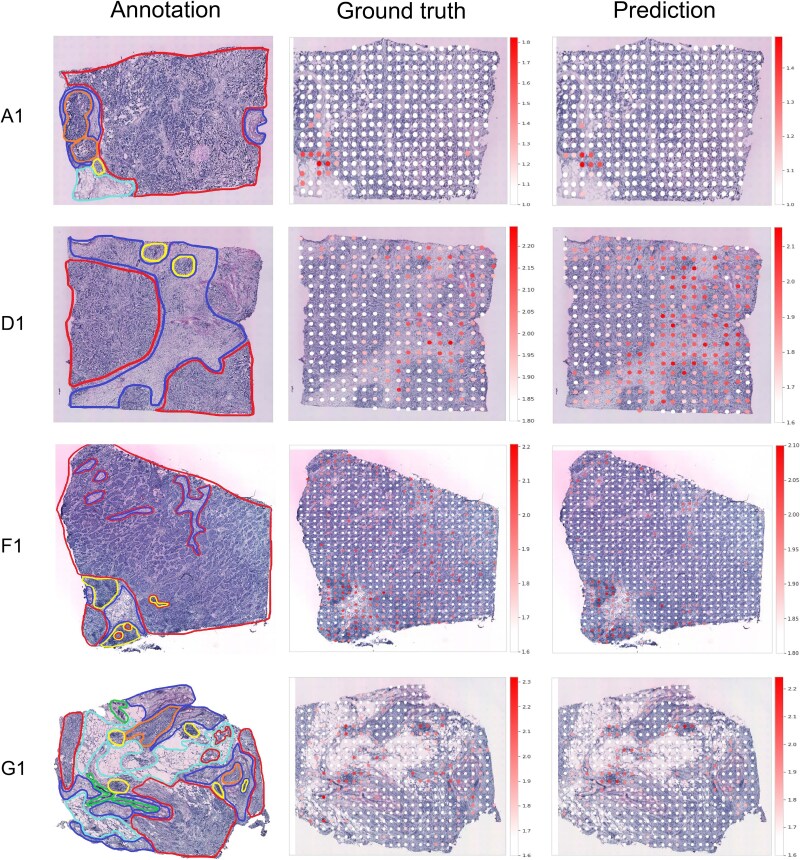
Spatial visualization of leukocyte mediated immunity gene set on HER2+ dataset. The samples in the first column are originally annotated by pathologists in the HER2+ dataset [20]. The first column contains pathologist annotations with tissue categories indicated in the legend: adipose, breast glands, in situ cancer, connective tissue, immune infiltrate, and invasive cancer. Ground-truth and predicted maps display the mean expression across genes in the set at each spot. Spots indicate overprediction, underprediction, or values within a predefined tolerance, with a shared scale across panels.

Based on pathway-enrichment analysis (supplementary results, Figure S10), we selected the leukocyte-mediated immunity gene set for spatial visualization, which is the enriched term with the highest coverage ratio. We mapped the set by averaging predicted expression across its genes and compared the result with pathologist annotations. Figure 5 shows that high expression regions align with the ground truth, particularly the yellow-marked immune-infiltrated regions on the annotated WSIs. This colocalization is consistent with the function of the leukocyte-mediated immunity gene set. A visualization for the amoeboid cell migration pathway, which is mainly enriched in tumor tissues, is provided in Fig. S11. Overall, these results illustrate SciSt’s ability to produce spatially faithful gene expression predictions. Our study demonstrates the use of predicted expression to map the spatial distribution of a specific function, suggesting potential for analyses of other function-related tissues.

### Biomarker genes of TLS on HER2+ dataset

**Figure 6:**
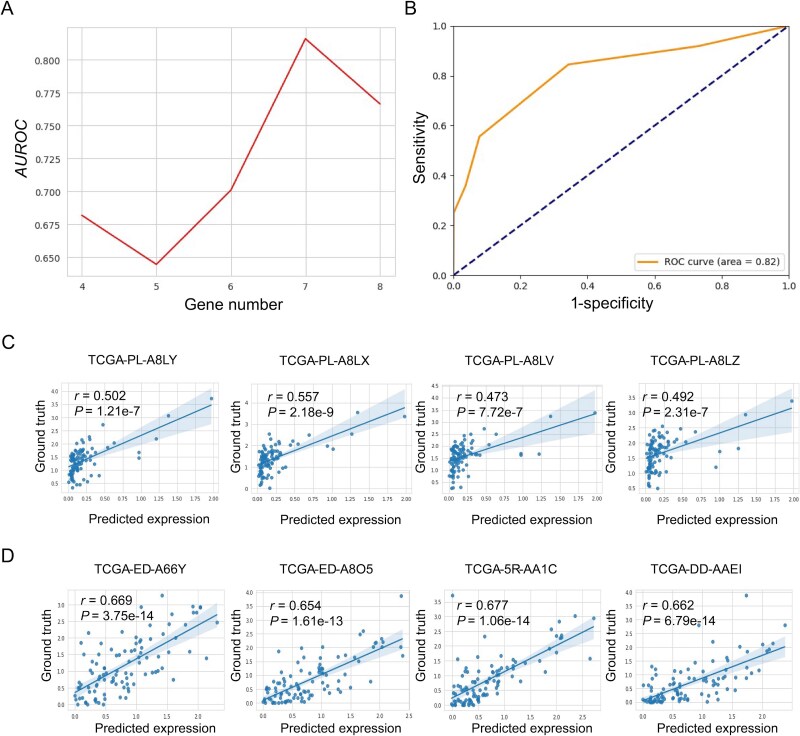
Screening of TLS biomarkers and external validation of SciSt. (A) Screening process of TLS biomarker. As the number of genes increases, AUROC gradually reaches a local peak. (B) The ROC curve for the seven selected genes. External validations of SciSt on TCGA-BRCA (C) and TCGALIHC (D) datasets with bulk RNA-seq data, respectively.

In addition, we derived TLS biomarkers on the HER2+ dataset using gene expression predicted directly from pathological images (rather than measured T- and B-cell expression as in prior work), enabling use where RNA profiling is unavailable. We trained a binary TLS classifier with predicted expression as features and varied the number of genes according to a data-driven ranking (supplementary methods). The cross-validated *AUROC* reached a local maximum at seven genes (Fig. 6A), with an *AUROC* of 0.82 (Fig. 6B). We therefore selected that seven-gene set as TLS biomarkers: *GNAS*, *IGHA1*, *C3*, *FASN*, *SCD*, *CD74*, and *BGN*.

### Generalization of SciSt in external bulk RNA-Seq datasets

To assess the generalizability and robustness of SciSt, we conducted external validation on bulk RNA-seq data from TCGA-BRCA and TCGA-LIHC datasets. The model was retrained by breast cancer and liver cancer ST datasets, respectively. For each cancer type, we randomly selected four specimens and computed their correlations between simulated bulk predictions and the corresponding bulk RNA-seq profiles. The simulated bulk predictions were generated by averaging the gene expression across all patches. Predicted and observed bulk expression showed significant positive correlations for all samples (Fig. 6C and D), indicating that SciSt generalizes across carcinomas and data modalities.

### Ablation experiments

**Figure 7:**
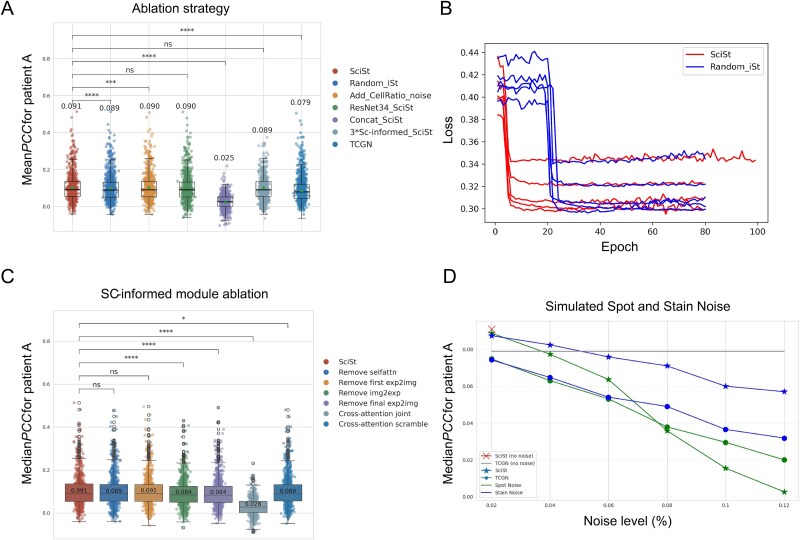
Results of ablation experiments. (A) Comparison results between SciSt, Random_iSt, Add_CellRatio_noise, ResNet34_SciSt, Concat_SciSt, 3*SC-informed_SciSt, and TCGN, evaluated by mean PCC on patient A. (B) Comparison of convergence epoch between SciSt and Random_iSt. (C) Target ablation study of SC-informed modules. Joint: cross-attention disabled via gating (cross_gate = 0). Scramble: cross-attention with randomized key/value permutations of tokens, breaking modality alignment while preserving cross-attention structure. (D) Performance of SciSt under simulated spot and stain noise at different noise levels.

We performed ablations with five variants: random_iSt, Add_CellRatio_noise, ResNet34_SciSt, concat_SciSt, and 3 × SC-informed_SciSt, trained identically to SciSt. Performance was evaluated on patient A using mean *PCC* and Wilcoxon signed-rank *P*-values (Fig. 7A).

To evaluate the effect of IE, we constructed Random_iSt by randomizing the noise representation. SciSt outperformed Random_iSt in both accuracy and convergence rate, while the latter showed an early 20 epochs plateau (Fig. 7B). The Add_CellRatio_noise variant perturbed the IE directly and achieved a *PCC* slightly lower than SciSt but higher than Random_iSt. In addition, reducing the single-cell reference size caused a statistically significant yet relatively small performance drop, indicating the robustness of SciSt to reference variation (Fig. S12). Modifying the image encoder (ResNet34_SciSt) or deepening the SC-informed block (3 × SC-informed_SciSt) did not result in noticeable performance degradation, indicating robustness to the image backbone and SC-informed depth. In contrast, Concat_SciSt exhibited a *PCC* decline from 0.091 to 0.025, highlighting the critical role of the SC-informed module. Overall, all variants except Concat_SciSt outperformed TCGN, demonstrating the effectiveness of the proposed backbone design.

Ablations further showed that removing individual components from the SC-informed module did not cause substantial *PCC* decreases, although removing either img2exp or the final exp2img module led to noticeable declines. When the entire cross-attention module was ablated, the joint configuration resulted in a much larger drop than scramble, indicating that the main performance gains arose from an intact cross-attention mechanism enabling coherent alignment and bidirectional feature fusion across modalities, while correct key–value alignment further enhanced accuracy.

For realistic noise, we inject varying proportions of spot and stain noise (Fig. 7D). SciSt remains stable within a moderate noise range, is more resistant to stain than spot noise, and still surpasses TCGN at 4% noise, demonstrating superior robustness. The training results using only high-predictivity genes as labels are shown in Table S5.

## Discussion

Here we introduce SciSt, a deep-learning model that predicts ST directly from WSIs using guidance from single-cell RNA-seq references. Across multiple cancer types, SciSt outperforms SOTA methods on quantitative benchmarks and in recovering spatial expression patterns. To our knowledge, this is the first WSI-to-ST approach that integrates real-world gene-expression data into model training. By enabling rapid and reliable ST inference from routine pathology images, SciSt reduces reliance on costly sequencing and accelerates disease-mechanism research.

STs provides rich biological insight but is limited by cost and scarce paired data, while large archives of routine H&E whole-slide images remain underutilized. SciSt adopts a biology-informed strategy: training is initialized with IEs computed from cancer type specific single-cell references. For each spatial location, an initial multigene profile is constructed as a weighted mixture of cell type compositions and used as a conditioning signal during learning. This shifts the focus from unconstrained end-to-end scaling to models guided by cellular priors and real expression supervision. The result is higher accuracy, clearer interpretability, and improved data efficiency and robustness, delivered in a modular pipeline that links pathology images to single-cell transcriptomics and unlocks the value of clinical image archives.

SciSt’s novelty lies in a prompt-conditioned architecture inspired by SAM, where biologically informed IEs serve as prompts that condition learning. An SC informed module applies cross attention to align image features with single cell references and to fuse morphology with expression. Ablations show that this module drives the gains. Removing individual subcomponents caused only small *PCC* losses, whereas a joint ablation that set all cross attention gates to zero and disabled interaction between streams produced a large drop. A scramble control that randomly permuted q, k, and v did not reproduce this decline. These results indicate that an intact cross attention architecture with coherent alignment and bidirectional fusion, rather than model capacity, underlies the performance.

SciSt supports several clinically relevant uses by turning routine H&E whole slide images into spatial gene-expression maps that align with pathologist-annotated immune-infiltrated regions, enabling microenvironment assessment within standard pathology workflows. Building on these predictions, a seven-gene TLS biomarker achieved an *AUROC* of 0.82 and can stratify patients when RNA profiling is unavailable. The model remains stable under simulated stain and spot noise and exceeds a strong baseline even at 4% noise, which supports integration into digital pathology pipelines where robustness is essential. By reducing reliance on additional sequencing and activating underused clinical image archives, SciSt lowers cost barriers and broadens access to spatial molecular analysis in real-world settings.

With rising compute, ever-larger WSI-to-ST models have appeared, yet scale alone rarely guarantees better accuracy or utility. In practice, growing parameter counts increase compute, memory, and data demands, which can hinder reproducibility and real-world deployment. Our results point to a more sustainable direction: targeted training guided by prior biological knowledge. When real single-cell expression is used as a training-time prior, even a lightweight framework like SciSt achieves strong performance and robustness. More broadly, the principle—coupling domain priors with multimodal learning as training “prompts”—is readily transferable beyond digital pathology to other image-to-omics mappings and cross-modal biomedical tasks. Rather than chasing model size, we advocate balancing capacity with data quality and priors to build models that are accurate, interpretable, and easier to operationalize.

Despite its contributions, this work has several limitations. First, the construction of the IE remains imprecise. It relies on publicly available single-cell reference data, and as more fine-grained, cancer- and cohort-matched atlases are released [33], this prior can become more informative and better guide the model. In addition, the current pipeline employs relatively coarse segmentation and a simplified, largely weighted-sum procedure for IE assembly; these choices can introduce boundary mixing and ignore factors such as cell size and mRNA abundance/distribution, potentially biasing the estimated signal. First, the precision of the IE remains improvable. It is currently constructed from publicly available single-cell reference data, and as more fine-grained, cancer- and cohort-matched atlases are released [33], this prior can become more informative and better guide the model. Second, the predictive scope is constrained by morphology. SciSt can reliably recover expression only for the subset of genes whose regulation leaves a discernible signature on histopathology, and the feature space obtainable from morphology alone is inherently limited. Third, broader evidence is needed to establish external validity and platform coverage: more publicly available STs datasets paired with high-resolution pathology images, as well as evaluations beyond Visium (e.g. MERFISH, Stereo-seq), are required to rigorously assess performance and generalizability.

In future work, we will prioritize improving IE precision by incorporating disease-specific single-cell reference panels, adopting finer-grained segmentation (e.g. CellViT [34] or CT-EMT [35]), and replacing the simple weighted-sum IE construction to better capture biological heterogeneity. Second, we will expand the predictable gene set by modeling gene–gene interactions, leveraging whole-transcriptome context, and transferring knowledge from readily predictable targets to those currently difficult to predict. Finally, we will validate SciSt across independent cohorts and platforms, incorporating domain adaptation to different scanners on newly released pathology-paired STs datasets.

## Conclusion

In conclusion, we present SciSt, a lightweight deep-learning framework to predict spot-level multi gene expression from histopathology. By initializing training with biologically informed IE, SciSt accelerates optimization, improves accuracy, and enhances interpretability. The model achieves SOTA performance on benchmark datasets and generalizes across cancer types and data modalities. Practically, SciSt enables estimation of STs directly from routine pathological images, reducing cost barriers and unlocking the latent value of clinical image archives. Conceptually, it demonstrates how embedding prior biological knowledge can substantially advance the scalability, robustness, and scientific utility of biomedical AI.

**Key Points**

- SciSt, a deep learning model, directly predicts spatial transcriptomics from pathological images and achieves state-of-the-art performance on two benchmark datasets.
- SciSt lowers the barrier to using STs, expanding its application scope.
- It incorporates single-cell reference data for the first time, bridging imaging and single-cell data through the Hover-Net model.
- SciSt highlights the importance of prior biological knowledge in biomedical AI.

## Supplementary Material

Supplementary_bbaf613

Pseudocode_bbaf613

SciSt_encoder

## Data Availability

The original spatial transcriptomics data used in this study can be accessed through the following links: (1) HER2+ dataset [https://github.com/almaan/her2st/]; (2) cSCC dataset [https://www.ncbi.nlm.nih.gov/geo/query/acc.cgi?acc=GSE144240]; (3) breast cancer spatial transcriptomics [https://data.mendeley.com/datasets/29ntw7sh4r/5]; (4) liver cancer spatial transcriptomics [https://www.10xgenomics.com/platforms/visium]; (5) PAAD dataset [https://github.com/mahmoodlab/hest/]. The single-cell datasets used in this study can be accessed through the following links: (1) HER2+ and breast cancer: GEO accession: “GSE176078”; (2) cSCC: GEO accession: “GSE144236”; (3) liver cancer: GEO accession: “GSE125449”; (4) PAAD dataset: “GSE242230” The external validation on TCGA can be found in https://portal.gdc.cancer.gov/.

## Code availability

The underlying code for this study is available in https://github.com/liyixin12139/SciSt.
