# Supplementary material for: SciSt: single-cell reference-informed spatial gene expression prediction from pathological images
Source: Brief Bioinform. 2025 Nov 20;26(6):bbaf613. doi: 10.1093/bib/bbaf613 (PMC12632197; doi:10.1093/bib/bbaf613)
Supplement: Supplementary_bbaf613 [file supplementary_bbaf613.docx]

**SciSt: single-cell reference-informed spatial gene expression prediction from pathological images**

*Yixin Li^1^, Fan Zhong^2^*, Lei Liu^2,3,4^**

1 Institutes of Biomedical Sciences, Fudan University, Shanghai, 200032, China

2 Intelligent Medicine Institute, Fudan University, Shanghai, 200032 China

3 Shanghai Institute of Stem Cell Research and Clinical Translation, Shanghai, 200120, China

4 Shanghai Institute of Infectious Disease and Biosecurity, Fudan University, Shanghai, 200032, China

# Methods

## Evaluation metrics

### Pearson correlation coefficients (*PCC*) for single gene

Although loss function could provide comparison results of models, it was not advisable to evaluate multiple gene expression prediction models by calculating the exact loss values of a single gene on each spot. We focused more on the spatial distribution of gene expression as it could emphasize biologically significant local regions on pathological images, which might contribute to a particular biological process. For each sample S with *n* spots, we could get the ground truth $\mathrm{gt}_{t}$ and prediction result $\mathrm{pred}_{t}$ of gene $t$ from SciSt, and they had the same shape $\mathbb{R}^{n}$. *PCC* for $S_{t}$ could be calculated by the following formula:

$${PCC}_{S_{t}}=\frac{cov(\mathrm{gt}_{S_{t}}, \mathrm{pred}_{S_{t}})}{\sigma\left( \mathrm{gt}_{S_{t}} \right)\times\sigma(\mathrm{pred}_{S_{t}})}$$

$S_{t}$ represents the expression of gene *t* on sample S. The mean and median *PCC* for each gene can also be calculated across all samples to serve as the overall performance.

### Dispersion scores higher than median *PCC*

The unpredictable genes led to a high variance of over *PCC* for each sample. We proposed the dispersion score to measure the discreteness of *PCC* higher than the median. The higher the dispersion scores, the better the spatial distribution consistency of genes upper than the median was:

$$Dispersion score=\sqrt{\Sigma_{t\in n}{({PCC}_{t}-{PCC}_{\mathrm{median}})}^{2}}$$

Here, *n* represents the number of genes with *PCC* higher than the median.

### Statistical test

**Single gene evaluation:** To evaluate the significance of the spatial distribution consistency, we introduced the positive correlation significance test for each gene. Specifically, if the distribution of gene expression across spots conformed to the normal distribution, the Pearson test would be applied, or the Spearman test would be used. Then, *P*-values were adjusted through the Benjamin-Hochberg method. By ranking the *P*-value of genes, we can get the most predictable genes to the unpredictable genes.

**Multiple models comparison:** For multiple-model comparisons, we applied paired Wilcoxon signed-rank tests to gene-level Pearson correlation coefficients (*PCC*s), conducted separately for each model’s mean and median. This nonparametric test was chosen because *PCC* distributions were non-normal and gene-wise observations were paired across models.

## Selection of biomarker genes for Tertiary Lymphoid Structures (TLS)

TLS refers to organized immune cell aggregates formed in non-lymphoid tissues. The previous study published the HER2+ dataset had constructed a TLS evaluation approach by high throughput sequencing and verified it by experiments. We built the categorical labels by dividing the above values into high and low TLS based on the median threshold. Then, we established a TLS classification model with gene expression predicted by SciSt. According to *P*-value sorting, the number of genes inputting to the model increased gradually, from the top 4 to all genes with *P*-value less than 0.05, guaranteeing biomarker genes had high reliability. The model was MLP, and the number of neurons was twice the number of genes. *AUROC* was employed to measure performance.

## External validation for bulk RNA-seq data in TCGA

To verify the generalizability of SciSt, we implemented external validation on two TCGA datasets. We randomly picked up samples whose WSI and RNA-seq data were derived from the same specimen. Then, WSIs were randomly cut into $224\times224$ patches with the proportion of background lower than 20%. These operations were accomplished using the Python package Histolab. The prediction results of patches belonging to the same sample were averaged to get the simulating bulk RNA-seq results, and the regression tests were implemented with the genes that had the top 100 *PCC* among all.

# Results

## Comparison with the Diffusion-Based Baseline Stem

We additionally evaluated the diffusion-based baseline Stem. Due to prohibitive compute (>50 hours to train a single HER2+ sample), we restricted experiments to cSCC (patient P2, 3 samples) and the PAAD dataset. Stem showed unstable performance—occasionally competitive but sometimes yielding negative *PCC* (Table S3). Moreover, Stem’s training and inference are extremely time-consuming, with detailed parameter comparisons reported in Table S4.

## Consistency of spots cluster results based on predicted multiple genes

We also investigated the expression pattern of predicted multiple genes at the spot level. During the implementation process, we simultaneously clustered the ground truth and predicted results for spots belonging to each model within a single sample. This approach ensured that spots assigned the same color displayed coinciding signatures. Consequently, different models had diverse labels and varying numbers of clusters.

SciSt’s multi-gene clustering results closely aligned with the ground truth on B4 and P2_rep2 (Fig S6, Fig S7), the samples with the highest median *PCC* in the HER2+ and cSCC datasets, respectively. We further focused on a localized region of samples for a detailed small-scale comparison, where the predictive advantages of SciSt became even more evident, indicating that the spot characteristics generated from SciSt predicted gene expression are reliable. The three labels on various models had differences, so it was difficult to distinguish the match level. To address this, we introduced *ARI* to unify standards. *ARI* is a commonly used clustering evaluation indicator with a value range between -1 and 1, where a value closer to 1 indicates greater consistency with the true label. Three *ARI*s were 0.226, 0.178, and 0.099 for SciSt, TCGN, and THItoGene, respectively on B4, and 0.237, 0.229, 0.026 on P2_rep, demonstrating high performance of SciSt.

## Functional analysis of accurately predicted genes

To assess whether SciSt preferentially predicts specific functional classes, we first assigned each gene to broad categories (immune, oncogenic, ECM/cell-structure, housekeeping) and compared *PCC* distributions across classes. No significant differences were detected, indicating no systematic functional-category bias in prediction performance (Fig S8).

We then defined well-predicted genes using FDR-adjusted *P*-value thresholds of 0.05 and 0.10 (to facilitate comparison with prior work). Across datasets, SciSt consistently yielded the largest numbers of well-predicted genes (Table S6). In the HER2+ dataset, the number of genes with adjusted *P-*values below 0.05 of SciSt achieved 80, 4.7 times that of TCGN with 17. This discrepancy increased to 47 times in the cSCC dataset. In addition, it is worth mentioning that there were 169 genes used for training in the cSCC dataset, of which 141 genes had *P-*values less than 0.05, representing the majority were predictable by SciSt.

Using these significant sets, we performed Gene Ontology (GO) functional enrichment analysis. Enrichment results of *P*<0.05 genes showed that GO terms with high confidence and gene ratio were concentrated on immune-related pathways on the HER2+ dataset, such as MHC family and antigen binding, similar to results in TCGN (Fig S9A). Further, to expand the number of enriched genes, the top enriched GO terms were mainly associated with the extracellular matrix, which had been reported to influence cellular morphology^[1]^ (Fig S9B). To some extent, it explained why our model could capture these highly predictable gene expressions. Besides, these related GO terms were not enriched in the other two models. Similar to the HER2+ dataset, GO terms enriched by genes with adjusted *P-*values below 0.05 and 0.1 on the cSCC dataset were also associated with cSCC, such as skin development and epidermis development related (Fig S9C, D).

Together, these results indicate that SciSt shows no overall preference for any functional category. The most accurately predicted genes in biologically coherent pathways that are plausibly captured by spatial or image-derived cues, with immune signatures in HER2+ and epidermal/ECM programs in cSCC.

## Single-cell reference Ablation experiments

To assess sensitivity to the single-cell reference, we replaced the default reference with one built from a single patient and with its random down-sampled versions (80%, 60%, 40%, 20%). As shown in Fig. S12, although some settings yielded statistically significant differences versus the original SciSt, the overall declines in mean *PCC* were modest, and all SciSt variants consistently outperformed TCGN. These results demonstrate that SciSt remains stable and robust to both the source and depth of the single-cell reference.

**Table** **S1. Public datasets used in our study**

| Cancer type | URL | Spots number | Single-cell dataset | Validation dataset |
| --- | --- | --- | --- | --- |
| Breast cancer (HER2+) | https://github.com/almaan/her2st/ | 11,548 | GSE176078 | Internal Validation |
| Breast cancer | https://data.mendeley.com/datasets/29ntw7sh4r/5 | 30,655 | GSE176078 | TCGA |
| Cutaneous squamous cell carcinoma (cSCC) | https://www.ncbi.nlm.nih.gov/geo/query/acc.cgi?acc=GSE144240 | 8,671 | GSE144236 | Internal validation |
| Pancrea cancer (PAAD) | https://www.ncbi.nlm.nih.gov/geo/query/acc.cgi | 22503 | GSE242230 | External validation |
| Liver cancer | https://www.10xgenomics.com/platforms/visium | 12,321 | GSE125449 | TCGA |

**Table S2. Definition of symbols in Methods**

| **Symbol** | **Definition** |
| --- | --- |
| **IE** | SC‑informed initial expression |
| $G_{\mathrm{ST}}$ | spatial gene expression from the ST dataset |
| $G$ | predicted genes |
| $\mathrm{SCR}$ | single-cell reference |
| $C$ | set of cell types，$C=\left\{ eoplastic, inflammatory, connective, non-neoplastic epithelial, \right.\left. \mathrm{dead} \right\}$ |
| $c_{i}$ | cell type, $c_{i}\in C$ |
| $N_{c_{i}}$ | number of cells of type $c_{i}\in C$ in the patch |
| $N_{total}$ | total cell count for a patch |
| $\mathrm{Ref}_{c_{i}}$ | gene-expression reference for $c_{i}$ |
| $N_{\mathrm{mc}}^{i}$ | number of single cells assigned to $c_{i}$ in the $SCR$. |
| $N_{G}$ | number of genes in $G$ |
| $\pi_{i}$ | weight for $c_{i}$, proportional to $N_{c_{i}}$ and normalized, so $\Sigma_{i}\pi_{i}=1$. |
| $d_{att}$ | attention dimension at the SC-informed module input |
| $X_{\mathrm{img}}$ | Input image patch |
| $N_{\mathrm{coord}}$ | number of spatial locations in the final CNN feature map, $N_{\mathrm{coord}}=H\times W$ (49 in our setting) |

**Table S3. Median *PCC* comparison between SciSt and Stem (diffusion-based model).**

| Datasets | Samples | SciSt | Stem |
| --- | --- | --- | --- |
| cSCC | P2_rep1 | 0.369 | -0.014 |
|  | P2_rep2 | 0.409 | 0.037 |
|  | P2_rep3 | 0.394 | -0.057 |
| Pancreas | TENX116 | 0.328 | 0.0806 |
|  | TENX126 | 0.274 | 0.356 |
|  | TENX140 | 0.341 | 0.567 |

**Table S4. Comparison of SciSt and Stem under same run configuration.**

| Models | Number of parameters | GPU memory | Training time | Inference time |
| --- | --- | --- | --- | --- |
| SciSt | 33.12M | 2948MiB | 3.22 Hours | / |
| Stem | 35.63M | 17024MiB | 221 Hours | 3.49 Hours |

Note: The comparison is complished by P2_rep1 sample on cSCC dataset. Number workers=10. SciSt does not need extra inference time.

**Table S5. *PCC* comparison between original and retrain results.**

| Dataset | Sample | Gene | Original *PCC* | *PCC* (*P* < 0.05 as label) |
| --- | --- | --- | --- | --- |
| HER2+ | C4 | *GNAS* | 0.740 | 0.691 |
|  | C4 | *FASN* | 0.666 | 0.707 |
|  | C4 | *IGHA1* | 0.652 | 0.601 |
|  | A2 | *C3* | 0.558 | 0.572 |
| cSCC | P2_ST_rep2 | *IMP4* | 0.455 | 0.452 |
|  | P2_ST_rep2 | *PTP4A2* | 0.408 | 0.411 |

**Table S6. Comparison of predictable gene numbers on the three models.**

| Dataset | Model | Number of predictable genes | |
| --- | --- | --- | --- |
|  |  | *P*<0.05 | *P*<0.1 |
| HER2+ | SciSt | 80 | 233 |
|  | TCGN | 17 | 70 |
|  | THItoGene | 0 | 0 |
|  | HisToGene | 0 | 0 |
| cSCC | SciSt | 141 | 162 |
|  | TCGN | 3 | 0 |
|  | THItoGene | 0 | 0 |
|  | HisToGene | 0 | 0 |


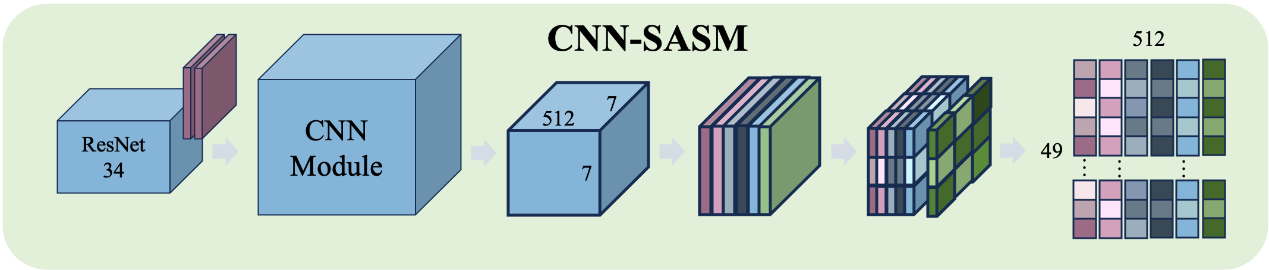
**Fig. S1 Architecture of CNN-SASM**

ResNet34 dropping the last two layers serves as the CNN module in CNN-SASM. The output of the CNN module goes through the channel self-attention module and spatial self-attention module sequentially. The final dimension is $B\times49\times512$ in our configuration, and *B* is the batch size.


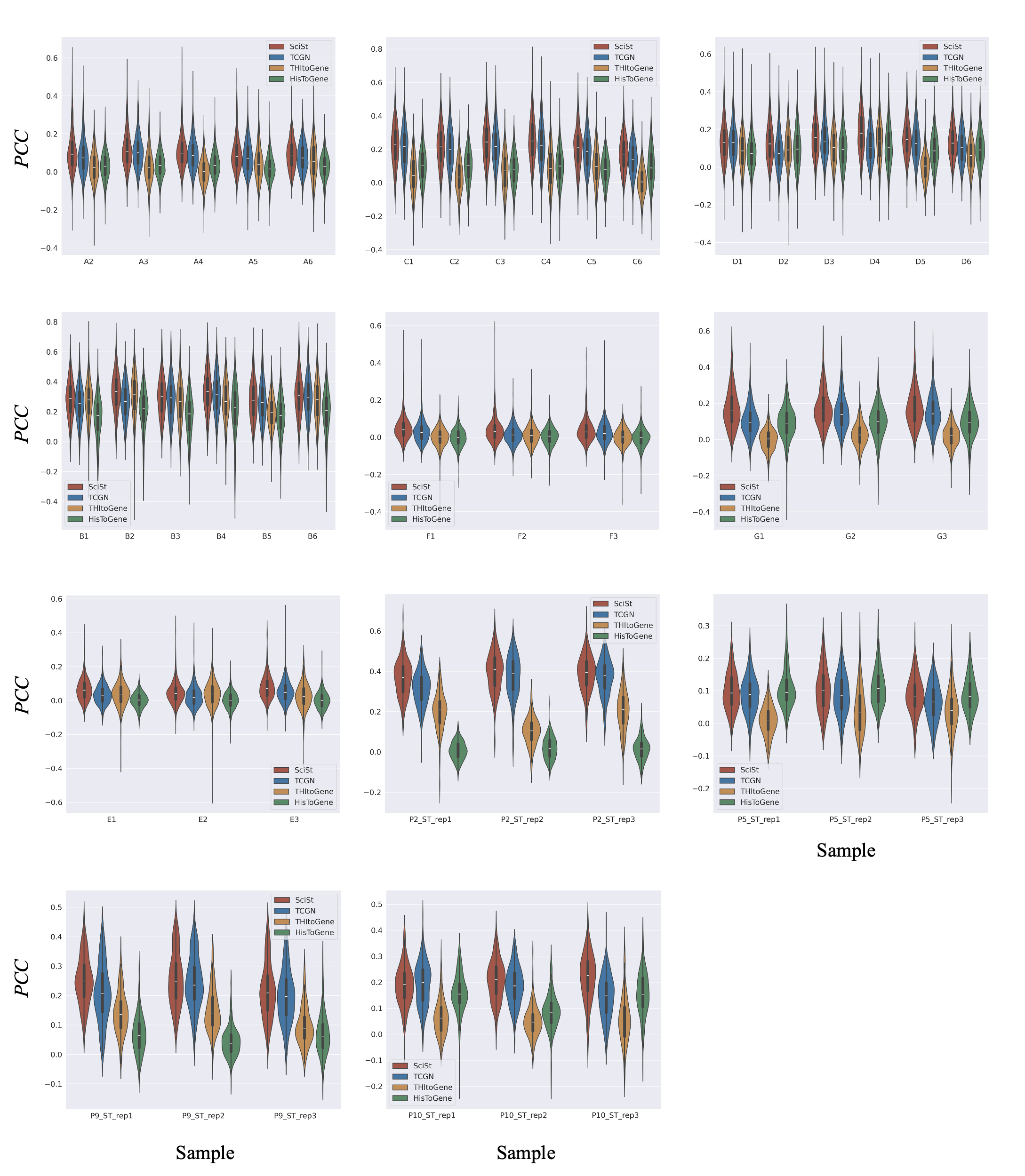


**Fig. S2 Violin plots for all patients on HER2+ and cSCC datasets**

Violin plots for each patient. They were drawn to compare the distribution of gene *PCC*.
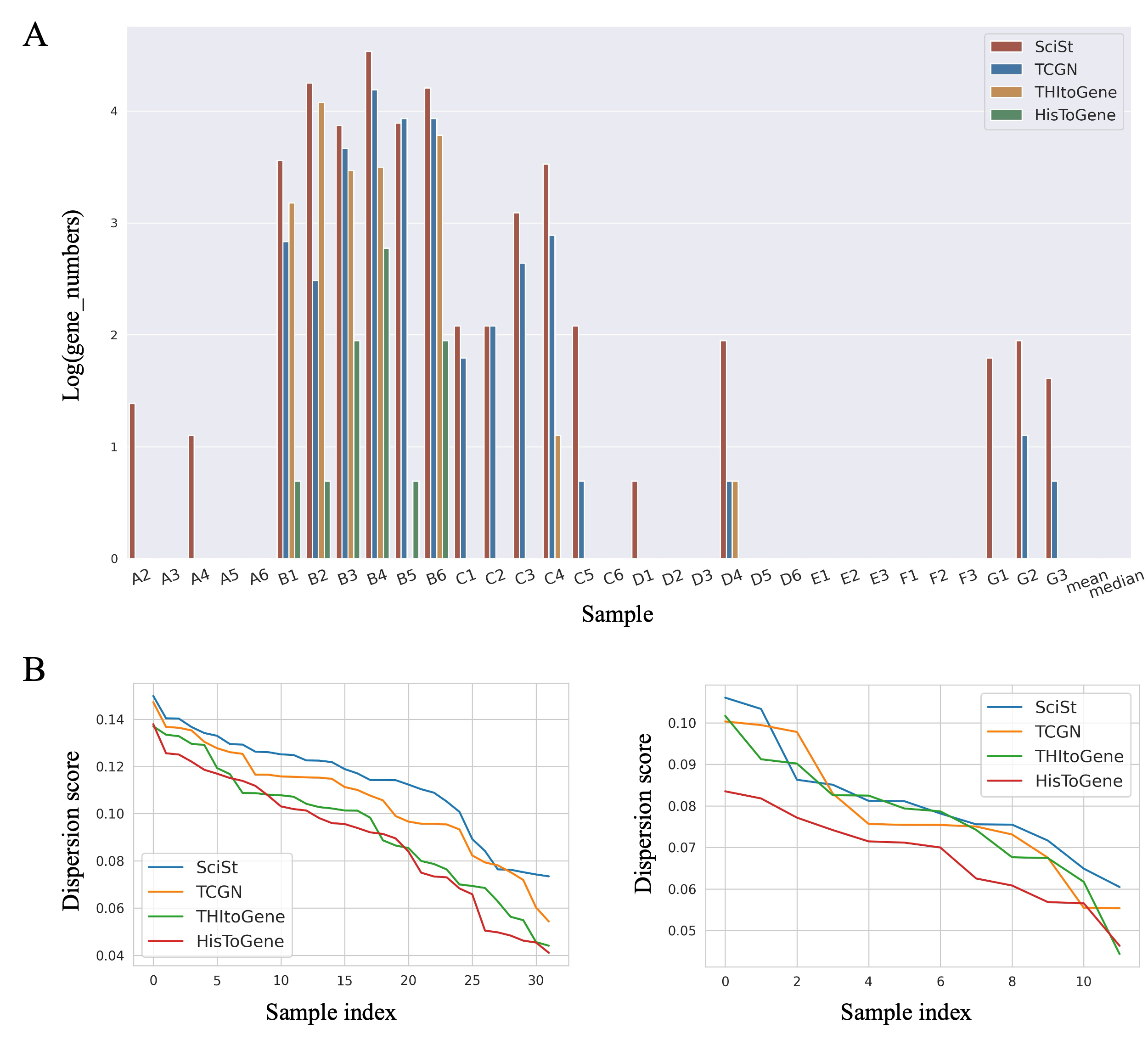


**Fig. S3 Performance comparison of three models**

**A** Number of genes whose *PCC* is higher than 0.5 across all samples on the HER2+ dataset. The logarithmic value of numbers was used as the ordinate to make the results easier to observe. **B** Dispersion score of three models on HER2+ and cSCC datasets. The samples were ordered according to the dispersion score.


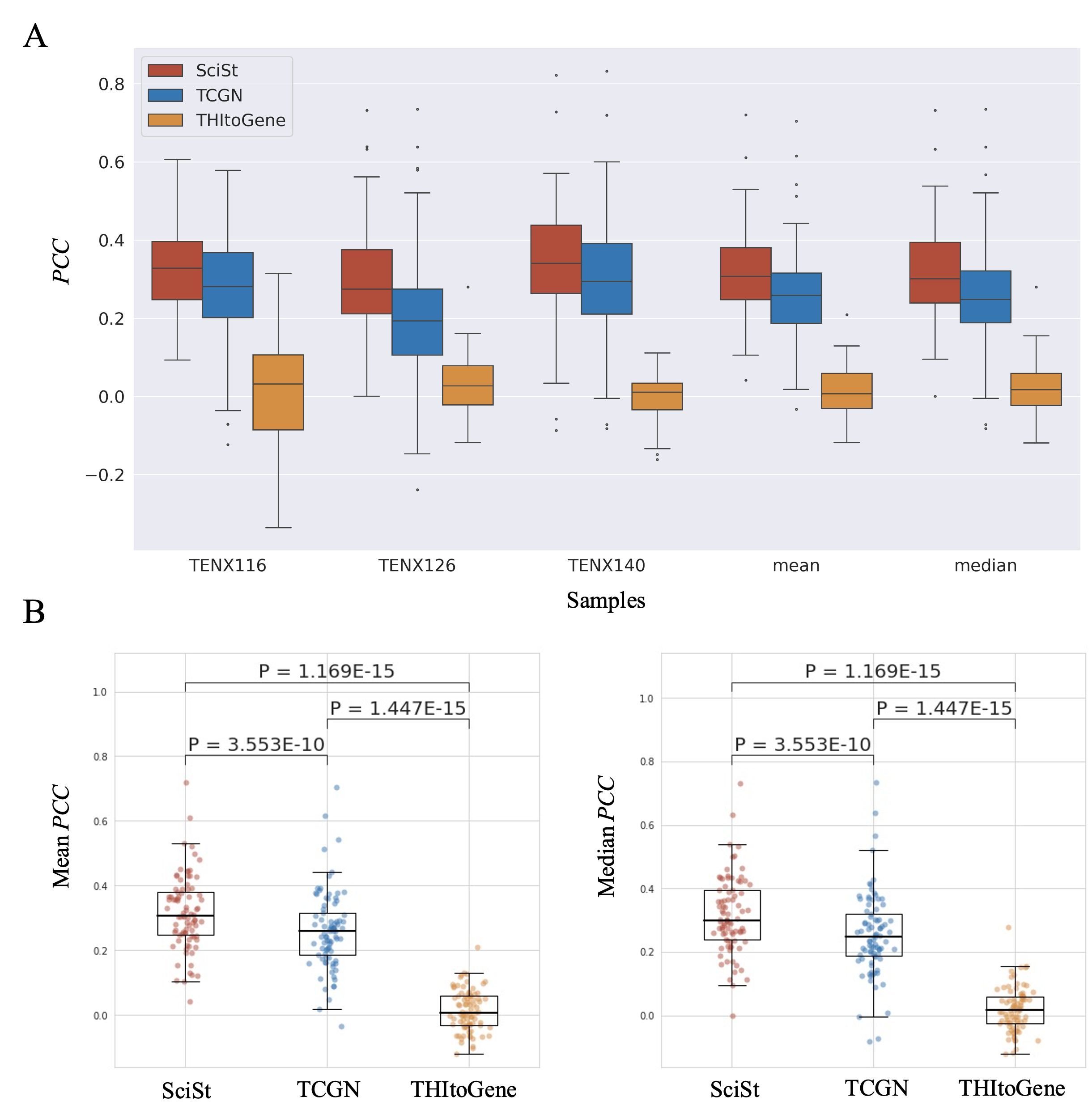


**Fig. S4 The performance of SciSt compared to TCGN and THItoGene on PAAD datasets**

A The comparison of SciSt with TCGN and THItoGene on the PAAD dataset. The evaluation indicator was the *PCC* of all genes belonging to the specific sample. Mean and median *PCC* were calculated across all samples to assess the overall performance of models. B Wilcoxon sign-rank tests for mean and median *PCC* of three models on the PAAD dataset.


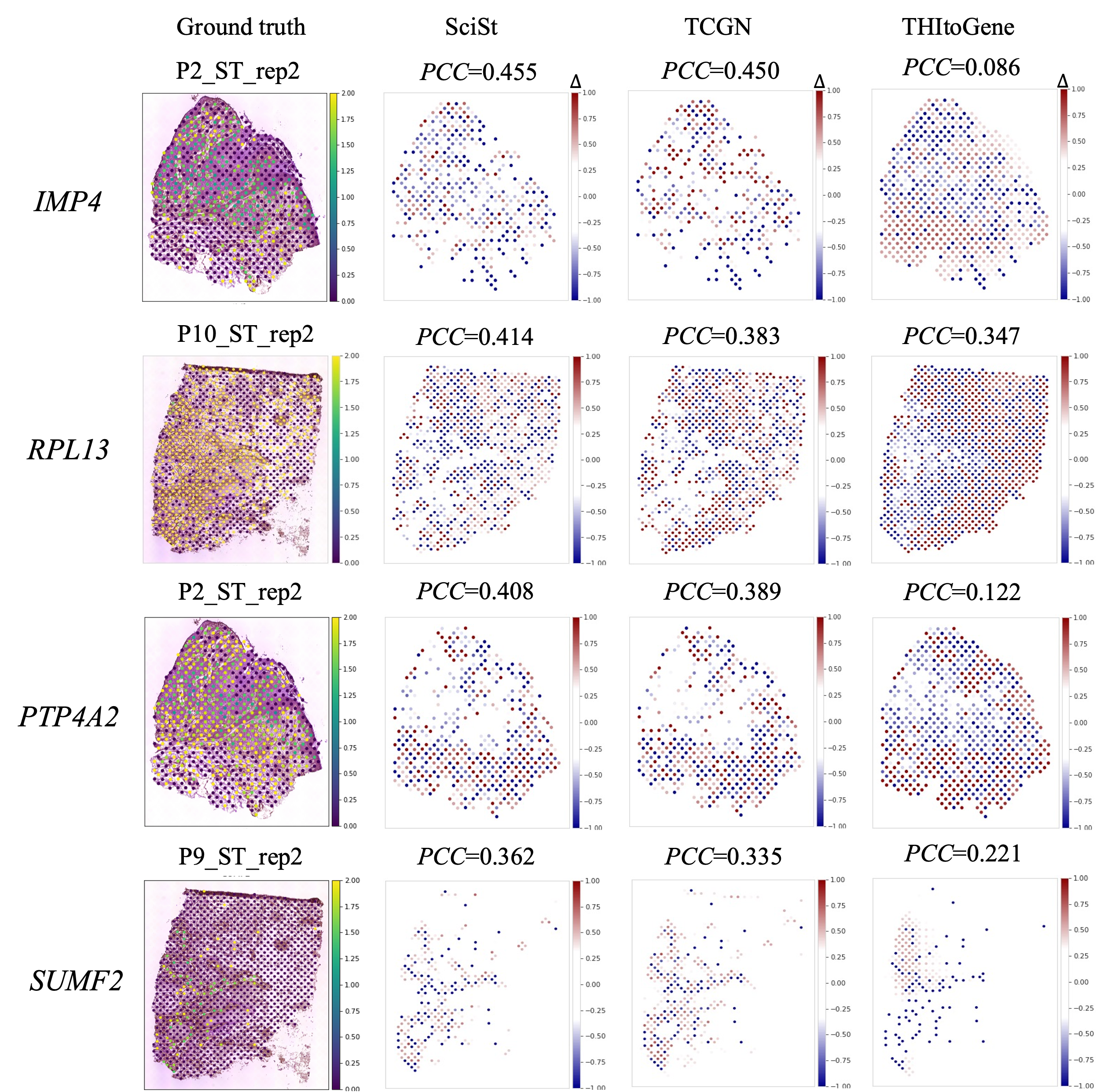


**Fig. S5 Spatial visualization of top accurately predicted genes on the cSCC dataset**

Top accurately predicted genes were selected according to the positive correlation significance tests, including *RPL13*, *SUMF2*, *PTP4A2*, and *IMP4*. We visualized the spatial distribution of the differences between these predicted gene expressions and the ground truths on the sample with the highest gene *PCC*. The red and blue spot indicates that the predicted expression is higher and lower than the ground truth, respectively, while the white points are within the acceptable range. All color bars are set to the same scale.


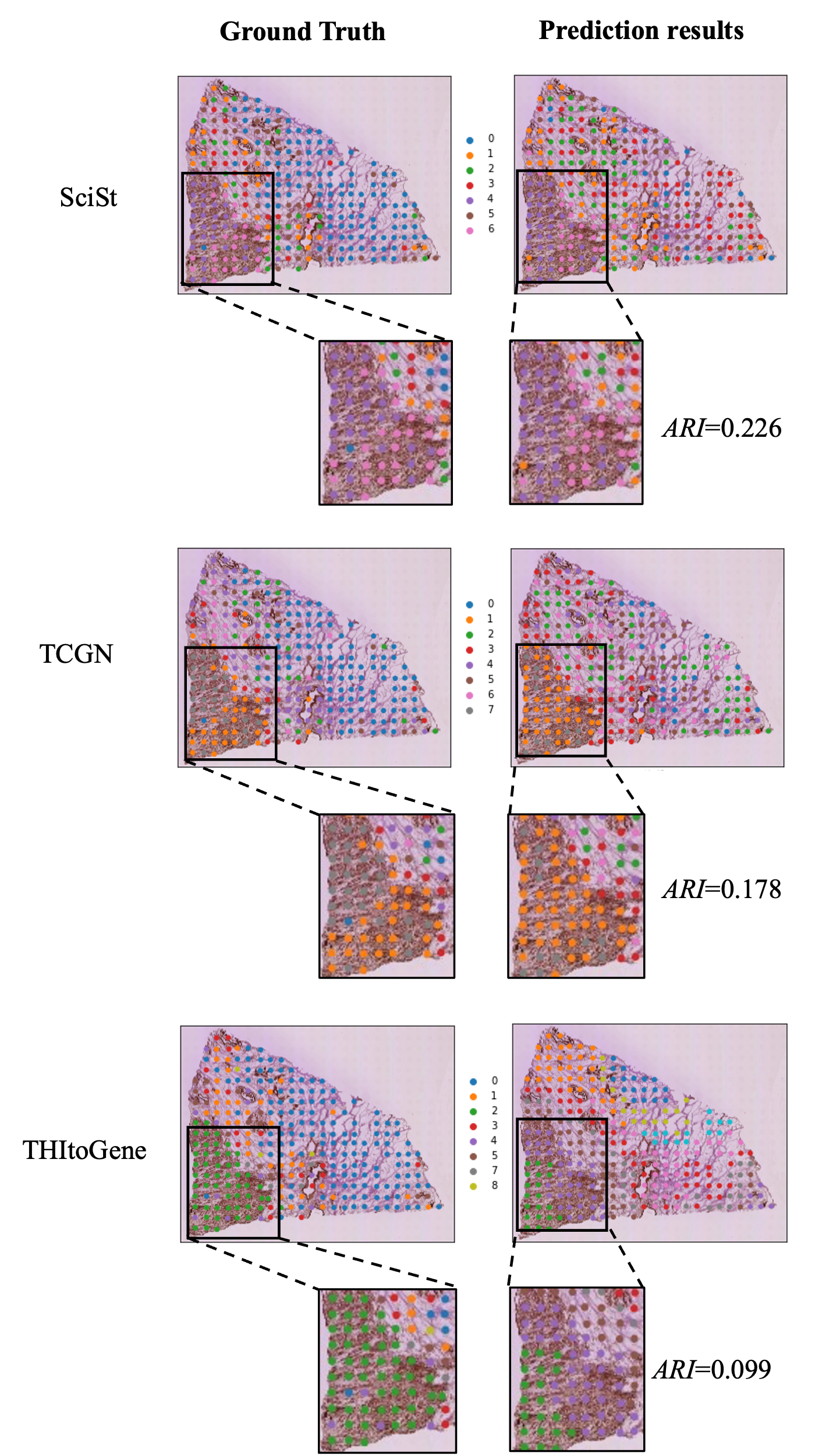


**Fig. S6 The consistency of multi-gene clustering between the predicted expression and the ground truth on three models**

Cluster results of three models on sample B4 belonging to the HER2+ dataset, which has the highest overall median *PCC*. The spots on ground truth and the predicted expression of each model were clustered together to ensure that spots with the same allocated color had similar characteristics. We further zoomed in on the local region for better comparison.


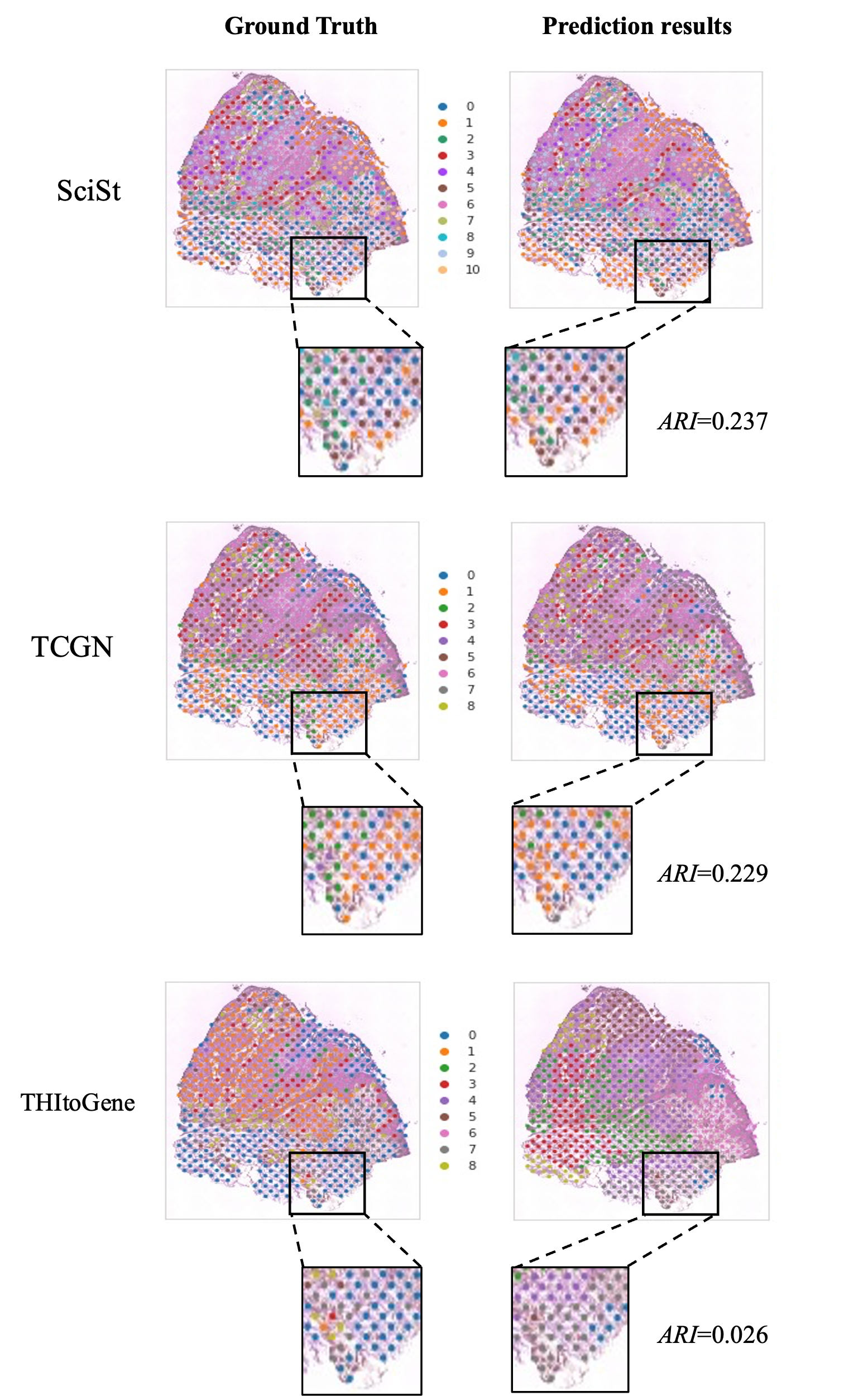


**Fig. S7** **The consistency of multi-gene clustering between the predicted expression and the ground truth on three models**

Cluster results of three models on sample P2_ST_rep2 belong to the cSCC dataset. The spots on ground truth and the predicted expression of each model were clustered together to ensure that spots with the same allocated color had similar characteristics. We further zoomed in on the local region for better comparison.


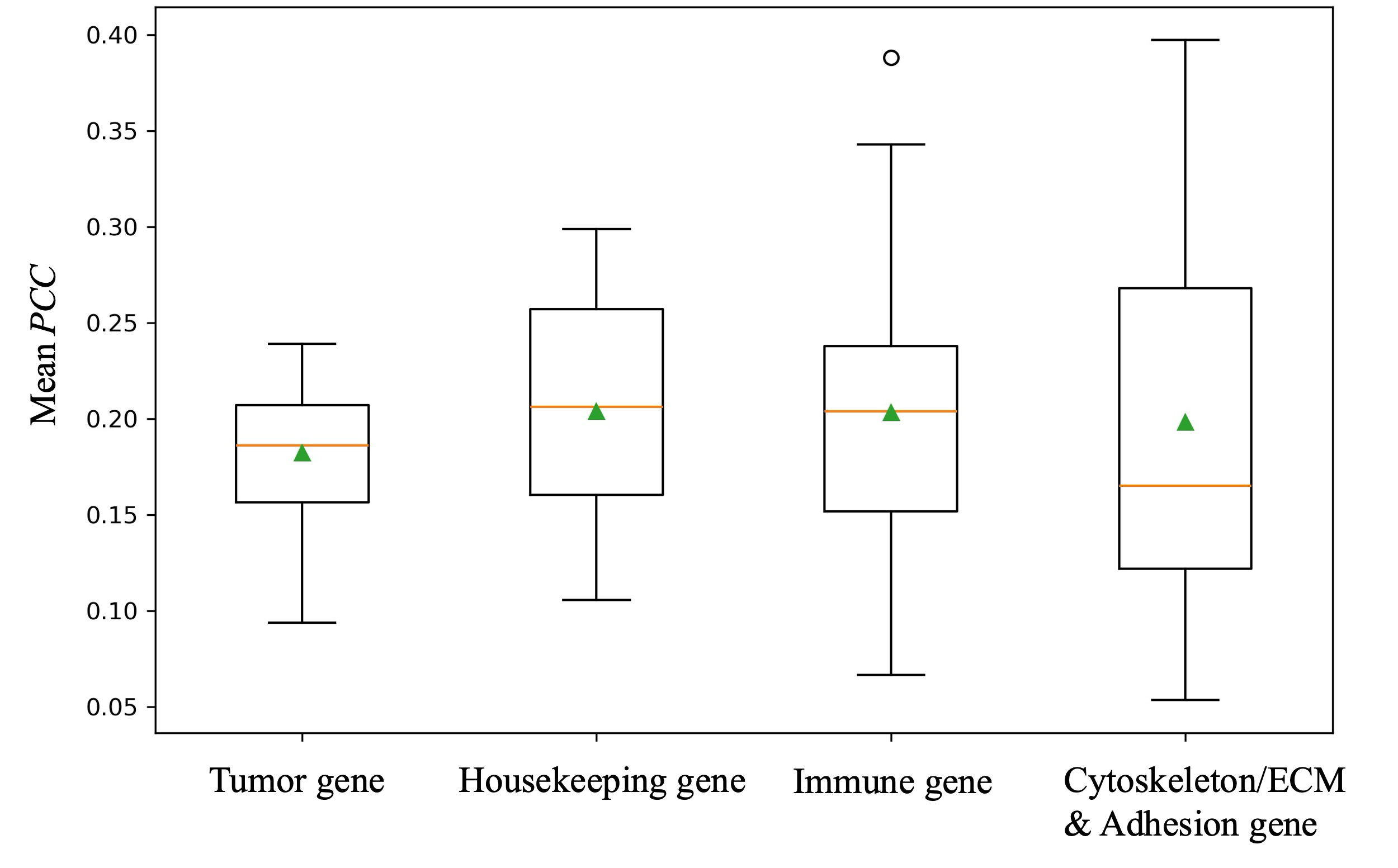


**Fig. S8 Distribution of Mean PCC Across Gene Categories**

This boxplot summarizes the distribution of mean *PCC* on HER2+ dataset for four gene categories (Tumor, Housekeeping, Immune, and Cytoskeleton/ECM & Adhesion). Boxes show the interquartile range (IQR). The orange line indicates the median and the green triangle the mean. Whiskers extend to 1.5 × IQR; open circles denote outliers.


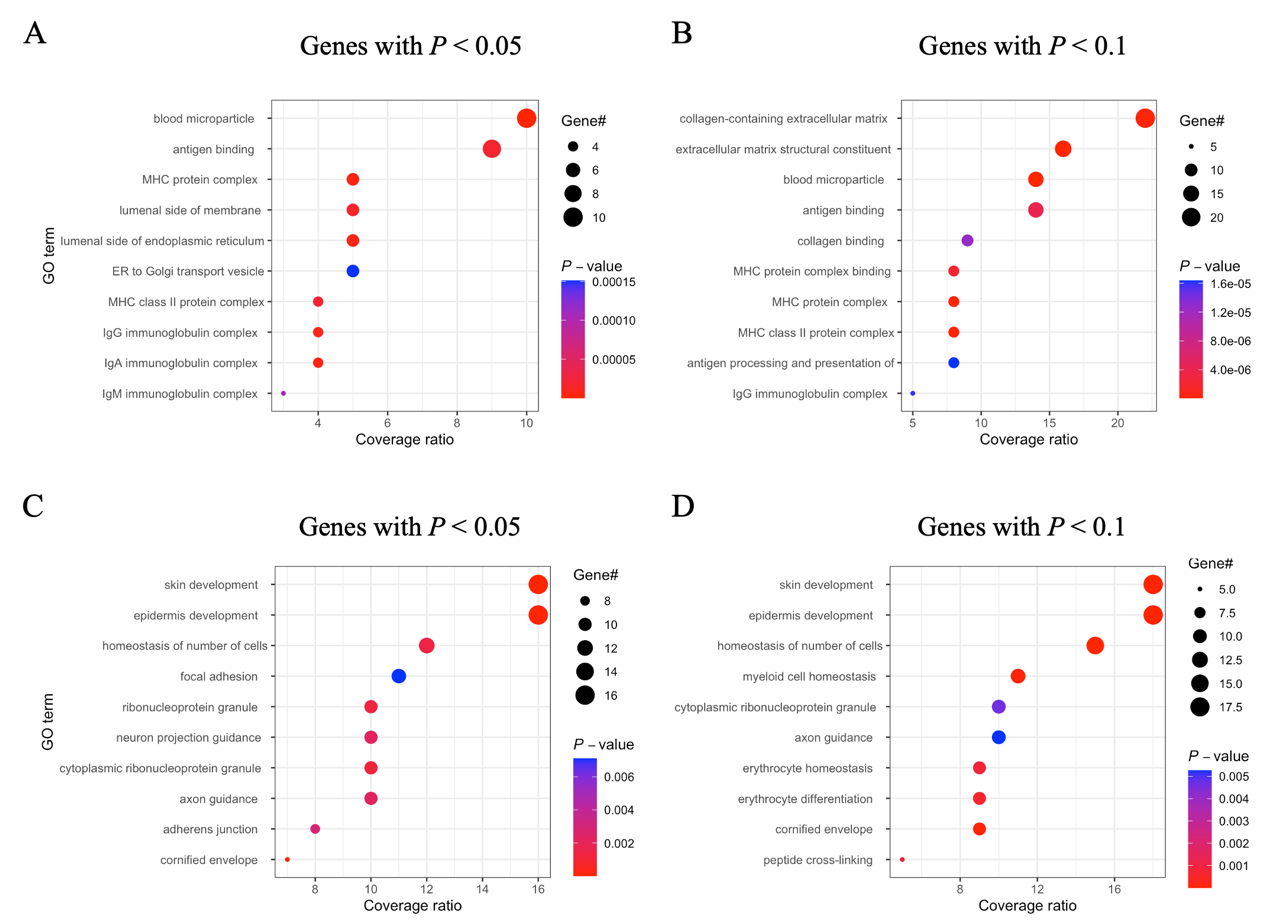


**Fig. S9 GO functional enrichment analysis of top accurately predicted genes**

Bubble plots of genes with *P*<0.05 (**A, C**) and *P*<0.1 (**B, D**). The values were calculated according to the average *P-*values of positive correlation significance tests across all samples on the HER2+ (**A, B**) and cSCC (**C, D**) datasets. The second threshold of *P* set to 0.1 is to be consistent with previous studies for comparison. The top 10 GO terms with high significance were displayed.


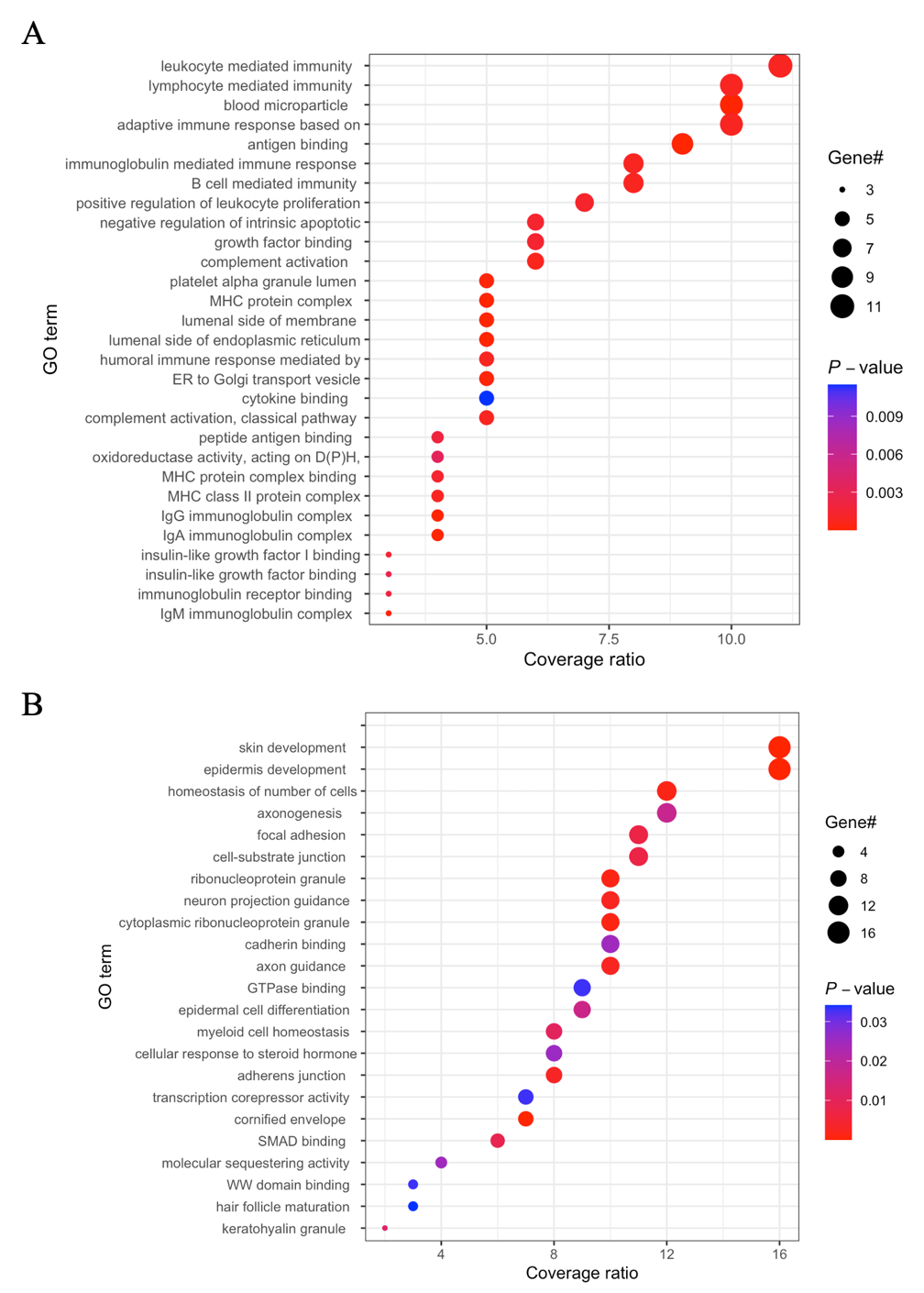


**Fig. S10 GO fuctional enrichment analysis**

**A** The top 10 GO terms with the high significance of GO_BP, GO_CC, and GO_MF categories on the HER2+ (**A**) and the cSCC (**B**) datasets, respectively.


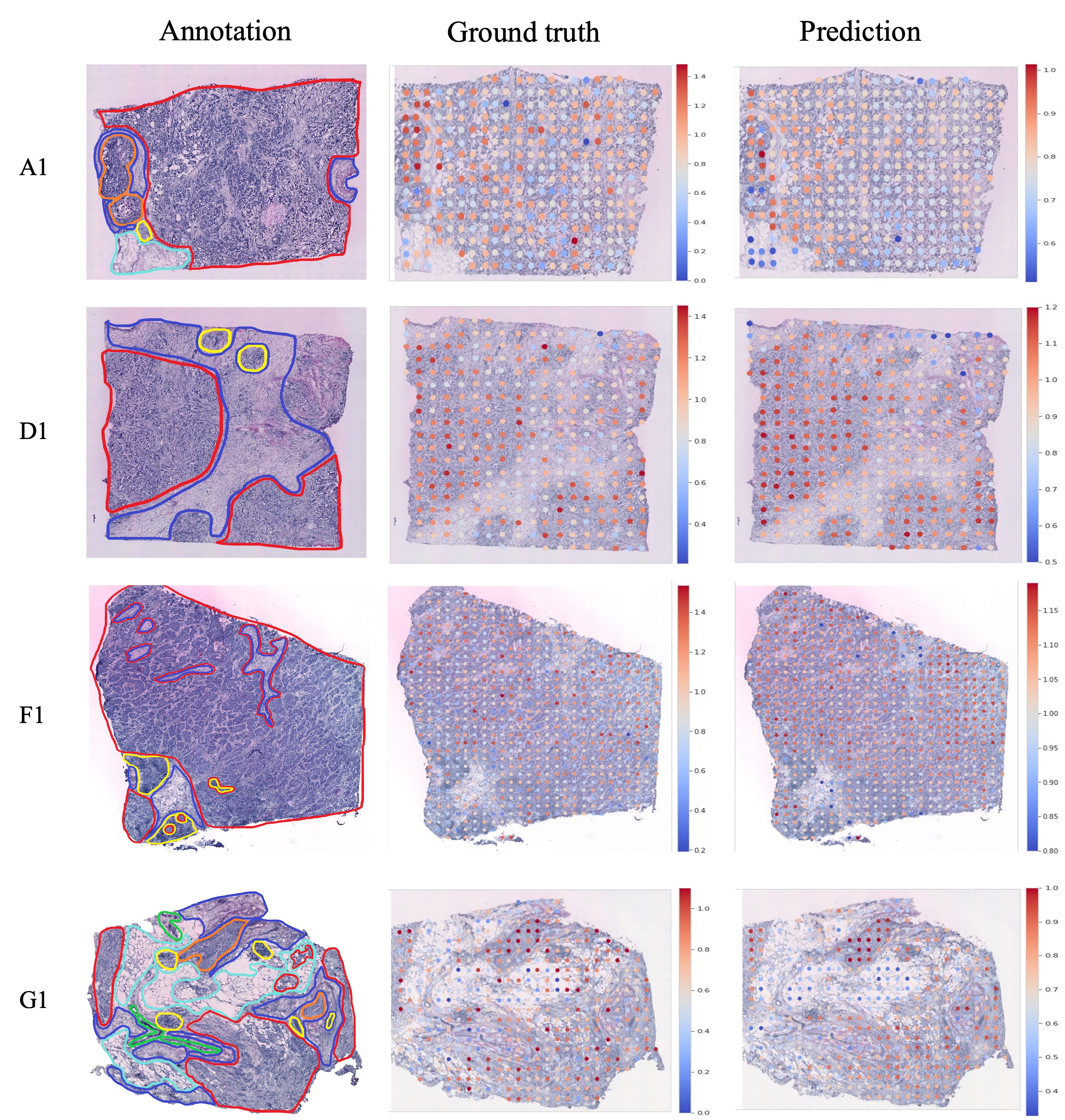


**Fig. S11 Spatial visualization of ameboidal-type cell migration gene set on HER2+ dataset**

The samples in the first column are originally annotated by pathologists in the HER2+ dataset. The color settings are as follows: adipose (cyan), breast glands (green), in situ cancer (orange), connective tissue (blue), immune infiltrate (yellow), invasive cancer (red). The red spots on ground truth and predicted expression samples were obtained based on the average of expressions belonging to the gene set.


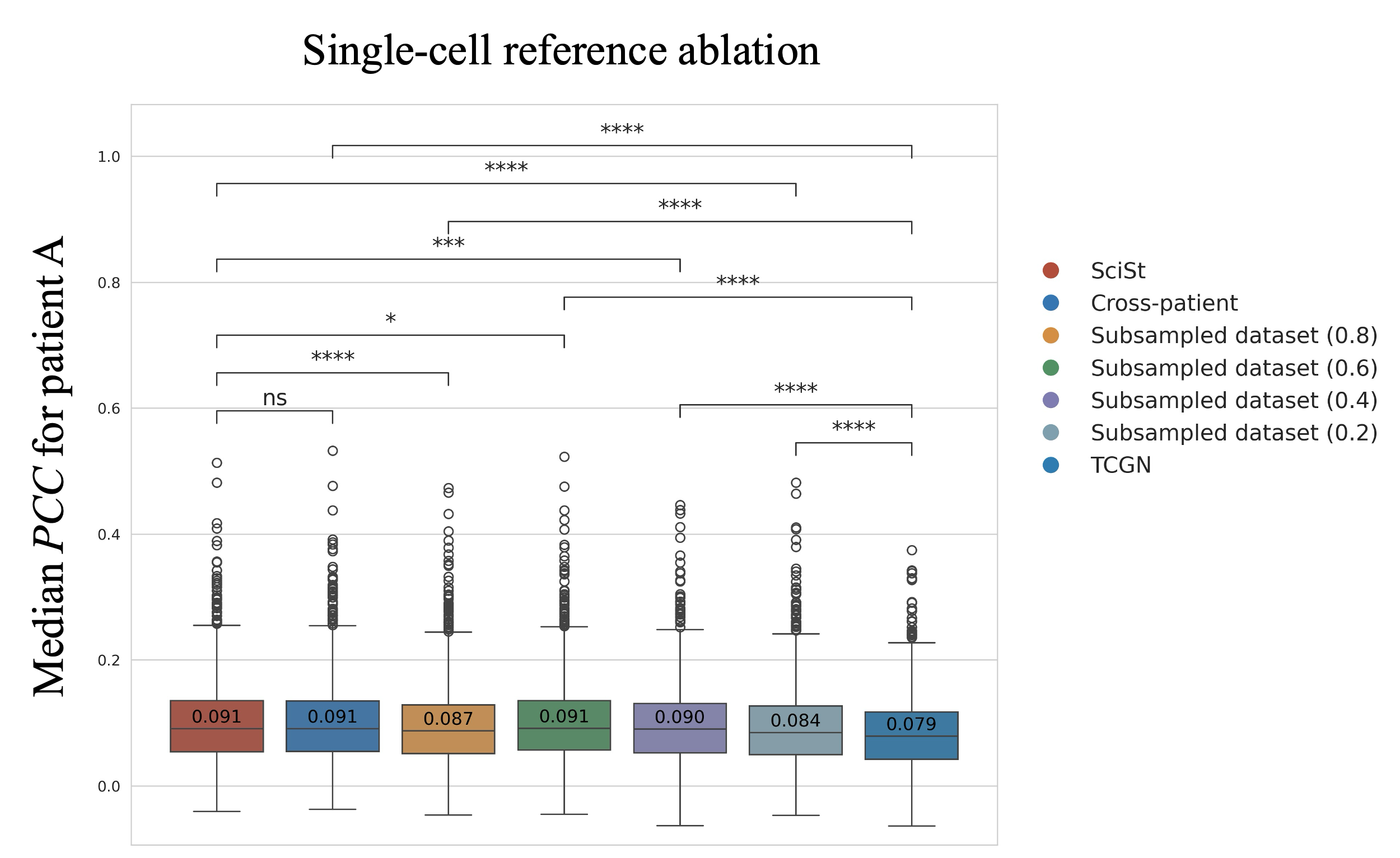


**Fig. S12 Single-cell reference ablation on patient A**

Median *PCC* for SciSt on patient A under different single-cell references: default SciSt reference, cross-patient reference, and subsampled references (80%, 60%, 40%, 20% of cells). TCGN is shown as a baseline. Statistical significance is assessed with Wilcoxon signed-rank tests across patient A samples.

**Reference**

[1] J. T. Ash, G. Darnell, D. Munro, et al. Joint analysis of expression levels and histological images identifies genes associated with tissue morphology. Nat. Commun. 2021;12:<https://doi.org/10.1038/s41467-021-21727-x>
