# Supplementary material for: SciSt: single-cell reference-informed spatial gene expression prediction from pathological images
Source: Brief Bioinform. 2025 Nov 20;26(6):bbaf613. doi: 10.1093/bib/bbaf613 (PMC12632197; doi:10.1093/bib/bbaf613)
Supplement: Pseudocode_bbaf613 [file pseudocode_bbaf613.pdf]

Algorithm 1: SciSt Encode

Input: P: image patches cut from the training pathological images; G_{ST}: high variance gene expression in the ST dataset for predicting gene G; Ref_i: reference genes for cell type c_i; f_{seg}: segmentation model; f_{SciSt}: SciSt model;

Output: y: predicted gene expression for G;

Initialize: $N \leftarrow$ Sizeof (training patches), $C \leftarrow$ Five cell types;

$G_{ST} \leftarrow \log(Normalization(\mathrm{G}_{ST}))$ `// Serve as labels`

for $s \in N$ **do**

    $N_{ci\_\_s} \leftarrow f_{seg}(P_s)$; `//` N_{ci} `is the number of cell type` ci

    $N_{total\_\_s} \leftarrow \sum_{c_i \in C} N_{c_i}$ `//` N_{total_s} `is the total cell number of` P_s

    $OrigExp_s \leftarrow \sum_{c_i \in C}(Ref_i \times \frac{(N_{c_i\_\_s})}{N_{total\_\_s}})$;

    $y \leftarrow f_{SciSt}(P_s, OrigExp_s)$;

    $s \leftarrow s + 1$; `// Increment s by 1`

end for
